# Membrane RRM2-positive cells represent a malignant population with cancer stem cell features in intrahepatic cholangiocarcinoma

**DOI:** 10.1186/s13046-024-03174-w

**Published:** 2024-09-06

**Authors:** Yongzhi Zhao, Shuting Xue, Danduo Wei, Jianjuan Zhang, Nachuan Zhang, Liping Mao, Niya Liu, Lei Zhao, Jianing Yan, Yifan Wang, Xiujun Cai, Saiyong Zhu, Stephanie Roessler, Junfang Ji

**Affiliations:** 1 The MOE Key Laboratory of Biosystems Homeostasis & Protection, Zhejiang Provincial Key Laboratory for Cancer Molecular Cell Biology, Life Sciences Institute, Department of General Surgery in Sir Run Run Shaw Hospital Affiliated to School of Medicine, Cancer Center, Center for Life Sciences in Shaoxing Institute, Zhejiang University, Hangzhou, Zhejiang Province China; 2 Shandong Cancer Hospital and Institute, Shandong Cancer Hospital of Shandong First Medical University, Jinan, Shandong Province China; 3 Institute of Pathology, Heidelberg University, University Hospital Heidelberg, Heidelberg, Germany; 4 Life Sciences Institute, Zhejiang University, 866 Yuhangtang Road, Hangzhou, Zhejiang Province China

**Keywords:** Intrahepatic cholangiocarcinoma, RRM2, Cell membrane localization, Tumor heterogeneity

## Abstract

**Background:**

Intrahepatic cholangiocarcinoma (iCCA) is one of the most lethal malignancies and highly heterogeneous. We thus aimed to identify and characterize iCCA cell subpopulations with severe malignant features.

**Methods:**

Transcriptomic datasets from three independent iCCA cohorts (iCCA cohorts 1–3, n = 382) and formalin-fixed and paraffin-embedded tissues from iCCA cohort 4 (n = 31) were used. An unbiased global screening strategy was established, including the transcriptome analysis with the activated malignancy/stemness (MS) signature in iCCA cohorts 1–3 and the mass spectrometry analysis of the sorted stemness reporter-positive iCCA cells. A group of cellular assays and subcutaneous tumor xenograft assay were performed to investigate functional roles of the candidate. Immunohistochemistry was performed in iCCA cohort 4 to examine the expression and localization of the candidate. Molecular and biochemical assays were used to evaluate the membrane localization and functional protein domains of the candidate. Cell sorting was performed and the corresponding cellular molecular assays were utilized to examine cancer stem cell features of the sorted cells.

**Results:**

The unbiased global screening identified RRM2 as the top candidate, with a significantly higher level in iCCA patients with the MS signature activation and in iCCA cells positive for the stemness reporter. Consistently, silencing RRM2 significantly suppressed iCCA malignancy phenotypes both in vitro and in vivo. Moreover, immunohistochemistry in tumor tissues of iCCA patients revealed an unreported cell membrane localization of RRM2, in contrast to its usual cytoplasmic localization. RRM2 cell membrane localization was then confirmed in iCCA cells via immunofluorescence with or without cell membrane permeabilization, cell fractionation assay and cell surface biotinylation assay. Meanwhile, an unclassical signal peptide and a transmembrane domain of RRM2 were revealed experimentally. They were essential for RRM2 trafficking to cell membrane via the conventional endoplasmic reticulum (ER)–Golgi secretory pathway. Furthermore, the membrane RRM2-positive iCCA cells were successfully sorted. These cells possessed significant cancer stem cell malignant features including cell differentiation ability, self-renewal ability, tumor initiation ability, and stemness/malignancy gene signatures. Patients with membrane RRM2-positive iCCA cells had poor prognosis.

**Conclusions:**

RRM2 had an alternative cell membrane localization. The membrane RRM2-positive iCCA cells represented a malignant subpopulation with cancer stem cell features.

**Supplementary Information:**

The online version contains supplementary material available at 10.1186/s13046-024-03174-w.

## Introduction

Intrahepatic cholangiocarcinoma (iCCA) is the second most common primary liver tumor after hepatocellular carcinoma (HCC) and one of the most lethal malignancies [1]. Over the past four decades, its incidence and mortality rates continue to increase world widely [1, 2]. Meanwhile, its overall five-year survival rate remains as low as 9%. As for therapy, tumor resection is available for patients with iCCA at a very early stage. However, the majority of patients (∼70%) are diagnosed with unresectable iCCAs. For these patients, the systematic chemotherapy is the standard treatment. The first-line systemic treatment was the combination of gemcitabine and cisplatin, which only allowed a median overall survival of < 1 year [3–5]. Recent progress in genomic landscape presented distinctive molecular characteristics of iCCA, revealing several oncogenic genomic alterations. Consequently, two types of targeted therapeutic agents, Ivosidenib and Pemigatinib, were generated and approved as the second-line therapy for patients with *IDH1* mutation and *FGFR* fusion, respectively [6–9]. Whereas, the drug resistance was frequently observed and the patient survival remained poor [10, 11]. Therefore, more efforts are needed to investigate molecular features of iCCA from various perspectives with the hope of developing novel therapeutic methods to improve patient outcomes.

iCCA is a highly heterogeneous tumor with different risk factors, histologic subtypes, a complex landscape of genomic and molecular alteration and distinct characteristics in the tumor microenvironment. Due to its heterogeneity nature, it is thus important to identify iCCA cell subpopulations with more malignant features and perform further in-depth studies to target these cells. Tumor initiating cells or cancer stem cells (CSCs) are thought to be responsible for tumor initiation, metastasis and recurrence, with the resistance to conventional chemotherapy and radiotherapy in many different cancers [12]. They possess the capacity of self-renewal, differentiation as well as generation of heterogeneous lineages in a bulk tumor, and represent a subpopulation with more malignant features in tumors. Although CSCs have been enriched in many other cancer types such as breast cancer, lung cancer and HCC etc., the studies of cancer stemness and CSCs in iCCA are very limited. Two recent studies revealed that NCAM^+^ c-kit^+^ iCCA cells might bear CSC features [13] and inhibitor of differentiation 3 (ID3) was revealed as a CSC-related factor in iCCA [14]. Further in-depth exploration and identification of CSC-enriched iCCA cell populations are needed.

Ribonucleotide reductase subunit M2 (RRM2), as the catalytic component of ribonucleotide reductase (RNR) complex, is essential for the maintenance of deoxynucleotide triphosphate (dNTP) pool homeostasis required for DNA replication and repair [15, 16]. RNR is reported as a cytoplasmic enzyme. Under genotoxic stress, it would translocate from the cytoplasm to the nucleus to maintain the dNTP level at DNA damage sites [17, 18]. In cancer, RRM2 plays important roles through its RNR-related enzymatic functions and its non-enzymatic functions. RRM2 was upregulated in multiple tumor types such as lung cancer. Its high level was associated with poor prognosis and aggressive characteristics of patients [19–24]. As the catalytic subunit of RNR complex, RRM2 was essential for promoting DNA replication and DNA damage repair by producing dNTP in cancer [21, 25]. RRM2 was also revealed to function with its non-enzymatic roles in tumor progression. For example, RRM2 activated the AKT pathway by directly binding to and stabilizing ANXA1 in renal cell carcinoma, and in HCC cells it sustained intracellular glutathione (GSH) by protecting glutathione synthetase (GSS) from degradation [26, 27]. However, the potential function of RRM2 in iCCA was unknown.

In this study, we established a global screening method and identified RRM2 as the top candidate with high expression in an iCCA subpopulation with malignant and stemness features and in iCCA cells positive for a stemness-reporter. Consistently, RRM2 silencing significantly suppressed iCCA malignancy phenotypes in vitro and in vivo. Moreover, an unreported cell membrane localization of RRM2 in iCCA cells was noticed and further thoroughly investigated. After experimental validation of RRM2 cell membrane localization, an unclassical signal peptide and a potential transmembrane domain were revealed for RRM2. Consequently, RRM2 trafficked to the cell membrane via the conventional endoplasmic reticulum (ER)–Golgi secretory pathway. Furthermore, the membrane RRM2-positive iCCA cells were sorted and they possessed CSC malignant features compared to the corresponding negative cells, revealing the tumor heterogeneity of iCCA from a new perspective.

## Materials and methods

### Clinical specimens and databases

A total of four iCCA cohorts and two HCC cohorts were used in this study (Table 1). iCCA cohort 1 included 91 iCCA patients with available paired tumor and non-tumor mRNA array transcriptome data (GSE76297). Cases in iCCA cohort 1 were from Thailand in Asia. iCCA cohort 2 included 36 CCA cases (31 iCCAs) with RNA sequencing data in all CCA tissues and 9 non-tumor tissues from The Cancer Genome Atlas (TCGA). Cases in iCCA cohort 2 were mainly Caucasian. iCCA cohort 3 included 255 iCCA patients with available RNA transcriptome data in their tumor tissues. Cases in iCCA cohort 3 were all Chinese [6]. iCCA cohort 4 consisted of 31 iCCA patients with available archived FFPE iCCA tissues. Cases in iCCA cohort 4 were from Shandong Cancer Hospital and Institute in China, and the institutional review board approved the use of these FFPE tissues and waived the requirement for informed consent.

**Table 1:** Summary of datasets and clinical specimens used in this study

Cohorts	Source	Cases	Datasets or Specimens
HCC Cohort 1	GSE76297	62 HCC cases	mRNA array (paired tumor & non-tumor tissues)
HCC Cohort 2	TCGA	371 HCC cases	RNA sequencing (tumor, n = 371; non-tumor, n = 50)
iCCA Cohort 1	GSE76297	91 iCCA cases	mRNA array (paired tumor & non-tumor tissues)
iCCA Cohort 2	TCGA	36 CCA cases (iCCA, n = 31)	RNA sequencing (tumor, n = 36; non-tumor, n = 9)
iCCA Cohort 3	*Cancer Cell, 2022*	255 iCCA cases	RNA sequencing (tumor, n = 255)
iCCA Cohort 4	Shandong cancer hospital	31 iCCA cases	FFPE tissues of iCCA cases, n = 31

HCC cohort 1 included 62 HCC patients with available paired tumor and non-tumor mRNA array transcriptome data (GSE76297). HCC cohort 2 included 371 HCC patients. mRNA sequencing data from 371 tumor tissues and 50 non-tumor liver tissues were used (The Cancer Genome Atlas, https://portal.gdc.cancer.gov).

### Cell culture and treatment

Human iCCA cell lines RBE and HUCCT1 were cultured in RPMI 1640 medium and human HCC cell line Huh7 were cultured in Dulbecco’s modified Eagle’s medium (DMEM), which were supplemented with 10% fetal bovine serum (Cat#FBS500-S, AusGeneX), 100 U/mL penicillin–streptomycin (Cat#15140–122, Gibco) and 1% L-glutamine (Cat#25030–081, Gibco). All cells were cultured in a humidified atmosphere of 5% CO_2_ at 37℃. RBE cells were from the Cell Bank of the Chinese Academy of Sciences (Shanghai, China). HUCCT1 and Huh7 cell lines were from Japanese Collection of Research Biosources Cell Bank (JCRB). Different dose of doxorubicin (0 μM, 0.5 μM,1 μM, 2 μM, 4 μM) (Cat# S1208, Selleck) was used as chemotherapeutic treatment for 18 h. Cells were treated with 20 μM MG132 (Cat# S2619, Selleck) for 8 h to inhibit the proteosome-related degradation, and with 100 nM Bafilomycin A1 (Cat# HY-100558, MedChemExpress) for 24 h to inhibit the lysosome-related degradation. Treatment with 15 μg/ml Brefeldin A (Cat# HY-100558, MedChemExpress) for 18 h and 2 mM Golgicide A (Cat# S7266, Selleck) for 15 h was used to block the transport of secreted and membrane proteins from endoplasmic reticulum to Golgi apparatus.

### Transfection of plasmids and siRNAs, stable cell line construction

For vector OS4-GFP reporter, the sequence of four concatenated repeats of a Oct4/Sox2 binding elements was synthesized and inserted into the AgeI/AseI sites of CMV-GFP. The whole length of RRM2 was amplified and inserted into NotI/XbaI sites of p3xflag-CMV-10 to generate vector Flag-RRM2. The whole length of RRM2 or RRM2 truncations were amplified through PCR and then inserted into NotI/XbaI sites of p3xflag-CMV-14 vector, to generate constructs RRM2-Flag, RRM2 Δ1-44(Δ1-44), RRM2 Δ1-38(Δ1-38) and RRM2 Δ223-246 (Δ223-246). Mutations of RRM2 VLA/DDD, RRM2 ALS/DDD, RRM2 double DDD (double DDD), RRM2 Δ42-44(VLA del), RRM2 Δ36-38(ALS del) and RRM2 12G (12G) were generated with the vector of RRM2-Flag and the ClonExpress MultiS One Step Cloning Kit (Cat# C113-02, Vazyme). All the sequences of the corresponding primers were shown in Table S1. The DNA template of β-catenin with mutations of Ser33 and Ser37 to Ala was kindly provided by Dr. Bin Zhao from our institute. The whole length of β-catenin and HA tag was amplified and inserted into PT3-EF1α vector using ClonExpress MultiS One Step Cloning Kit.

RRM2 siRNAs, CTNNB1 siRNAs and scramble negative control siRNAs were purchased from GenePharma Co., Shanghai, China. The detailed information for siRNA targeting sequences were listed in Table S1.

Lipofectamine 2000 Reagent (Cat# 11,668,019, Invitrogen, US) was used for transfections of plasmids and Rfect siRNA Transfection Reagent (Cat# 11,011, BIOTRAN) was used for transfections of siRNAs.

For generation of OS4-GFP/CMV-GFP RBE cell line, RBE cells were transfected with pOS4-GFP or pCMV-GFP using Lipofectamine 2000 Reagent and then selected with 500 μg/mL G418 for 10 days and the resulting mixed cell colonies were maintained with 200 μg/mL G418. For generation of OS4-GFP/CMV-GFP HUCCT1 cell line, HUCCT1 cells were transfected with pOS4-GFP or pCMV-GFP using Lipofectamine 2000 Reagent and then selected with 200 μg/mL G418 for 5 days and the resulting mixed cell colonies were maintained with 50 ~ 100 μg/mL G418.

### Tumorigenicity assay in BALB/c nude mice

All mice experiments were approved by the Experimental Animal Committee of Zhejiang University. All animal experiments met the Animal Welfare Guidelines. BALB/c nude mice were purchased from Shanghai SLAC Laboratory Animal Co.Ltd. All mice were housed in Zhejiang University Laboratory Animal Center in laminar-flow cabinets under specific pathogen-free conditions at room temperature with a 24-h night-day cycle.

For the tumorigenicity assay in Fig. 2G-H, HUCCT1 cells were transfected with siCtrl or siRRM2. 48 h after transfection, cells were suspended in 1X PBS, and then mixed with Matrigel (1:1) (Cat# 354,284, Corning) or without Matrigel. They were then injected subcutaneously into the flanks of 5-week-old male BALB/c nude mice. 10,000 cells were injected per site. Five mice for each group (siCtrl, siRRM2#1 and siRRM2#2) were used. For the tumorigenicity assay in Fig. 6E, 5-week-old male BALB/c nude mice were randomly divided into six groups (RRM2^+^ 10,000 cells, RRM2^+^ 1,000 cells, RRM2^+^ 100 cells, RRM2^−^ 10,000 cells, RRM2^−^ 1,000 cells, RRM2^−^ 100 cells). Four mice were used for each group. The sorted RRM2^+^ and RRM1^−^ HUCCT1 cells were suspended in 1X PBS, and then mixed with Matrigel (1:1). The indicated number of cells were injected subcutaneously into the flanks of BALB/c nude mice. Tumor formation was monitored twice a week. Tumor size was measured and calculated by the formula of “$\text{Volume}= 0.5 \times {\text{Width}}^{2}\times \text{Length}$”.

### Cell viability assay, colony formation assay and migration assay

Cell viability was detected using 3-(4,5-dimethylthiazol-2-yl)-2,5-diphenyltetrazolium bromide (MTT, Cat# 298–93-1, Sangon Biotech) assay. Briefly, the corresponding RBE (1000 cells/well) or HUCCT1 (1000 cells/well) cells were seeded in 96-well plates and cultured for 5 days. Cell viability was measured each day. For chemoresistance detection assay, RBE (4000 cells/well) or HUCCT1 (5000 cells/well) cells were seeded in 96-well plates and exposed to doxorubicin at the indicated concentrations. Cell viability was measured at 24 h upon the doxorubicin incubation.

For colony formation assay, RBE (700 cells/well) or HUCCT1 (700 cells/well) cells were seeded in 6-cm dishes and cultured for 12 days. Colonies were then fixed with methanol, stained with crystal violet and counted.

For migration assay, 3 × 10^4^ cells were seeded into the upper transwell chambers (Cat# 353097, Falcon) of 24-well with uncoated 8-µm pores in serum-free medium. Complete medium for cell culture was added to the lower chambers as a chemoattractant. After 36 h of incubation, cells remaining on the upper surface of the membrane were removed with a cotton swab, and cells that invaded through the membrane filter were fixed with 100% methanol, stained by crystal violet, and photographed under a microscope. The number of invading cells was manually counted per high-power field for each condition and six fields on each membrane were randomly selected.

### Spheroid formation assay

For spheroid formation assay, 500 HUCCT1 cells were plated in Ultra Low Attachment 24-well plates (Cat# 3473, Corning Incorporated Life Sciences) and cultured in 1640 medium/F12 (Invitrogen) supplemented with B27 (Cat# 17,504,044, Gibco), 1 μg epidermal growth factor (Cat#AF-100–15, PeproTech), 1 μg basic fibroblast growth factor (Cat# 100-18B, PeproTech). Cells were incubated in a CO_2_ incubator for 14 days, and spheroids with diameter ≥ 50 μm were counted under a microscope.

### Flow cytometry and cell sorting

Cultured cells were collected by trypsinization and cell pellets were washed with PBS prior to resuspension in PBS with 0.5% fetal bovine serum. For OS4-GFP reporter system, cells were trypsinized and washed with PBS. For RRM2 detection, the trypsinized and washed cells were incubated with anti-RRM2 (ab57653, Abcam) (1 µg/1 × 10^6^ cells) for 30 min at room temperature. Then the secondary antibody Alexa Fluor ® 488 goat anti-mouse IgG (H + L) (ab150113) was used at 1/2000 dilution for 30 min at room temperature. Next, flow cytometry was then done on Beckman CytoFlex, and data were analyzed using FlowJo software (Tree Star).

For fluorescence-activated cell sorting (FACS), cells were treated as above and sorted using a BD FACSAria II (BD Bioscience). The top 5% strongly stained cells were referred to as positive cells, and the bottom 5% stained cells were sorted as negative cells.

### Mass Spectrometry (Mass Spec) and Flag immunoprecipitation (Flag-IP)/Mass Spec

For mass spectrometry of OS4-GFP^+^ and OS4-GFP^+^ RBE cells, about 200,000 of OS4-GFP^+^ cells and the same amount of OS4-GFP^+^ cells were sorted from a total of 10^7^ RBE iCCA cells. The sorted cells were pelleted and sent for mass spectrometry analysis in our institute.

For Flag-IP and mass spectrometry, cells were transfected with RRM2-Flag. 48 h after transfection, cells were collected and lysed in IP buffer. The lysates were then incubated with anti-Flag-M2 magnetic beads (Cat# M8823, Sigma–Aldrich) at 4 °C for 4 h. The immunoprecipitated proteins were subjected for SDS-PAGE and the SDS-PAGE gel was then stained with Coomassie brilliant blue G250. The 44 kDa RRM2 was carefully sliced out and sent for mass spectrometry analysis in our institute.

### Protein extraction, western blot

Cells were lysed in IP buffer (1% NP40, 150 mm NaCl, 50 mM tris PH 7.4, 10% glycerol) on ice for 30 min, then centrifuged at 12,000 rpm for 20 min to collect the supernatant as cell lysates. Cell lysates were then separated by SDS-PAGE Gel and transferred to PVDF membranes. The membranes were incubated with indicated primary antibodies and then secondary antibodies conjugated to horseradish peroxidase for enhanced chemiluminescence detection of the signals. These antibodies included anti-RRM2 (Cat#HPA056994, Merck), anti-Flag-M2 (Cat#F3165, Sigma), anti-OCT4 (Cat#311,263–1-AP, Proteintech), anti-SOX2(Cat#R1106-1, Huabio), anti-Nanog (Cat#67,255–1, Proteintech), anti-β-catenin (Cat#66,379–1, Proteintech), anti-EGFR (Cat#ET1604-44, HuaBio), anti-E-cadherin(Cat#24E10,CST), anti-Histone H3(Cat#A2348, Abclonal), anti-Tubulin (Cat#D3U1W, CST), HRP-linked anti-rabbit IgG antibody (Cat#309–035-003, Jackson Immuno Research), HRP-linked anti-mouse IgG antibody (Cat#209–035-082, Jackson Immuno Research).

### Cell fractionation assay

The cytoplasm and cell membrane fraction were separated by sucrose gradient fractionation as previously [28]. Three 10 cm dishes (~ 10^7^ cells) were prepared and rinsed with ice cold PBS. Cells were then harvested with 1 ml per dish of sucrose buffer (250 mM Sucrose, 20 mM HEPES,10 mM KCl, 1.5 mM MgCl2, 1 mM EDTA, 1 mM EGTA, 1 mM DTT, 1 × protein inhibitor and phosphatase inhibitor, pH 7.4) on ice using a cell scraper. They were lysed adequately on ice for 30 min and the lysates were centrifuged at 720 g at 4 °C for 5 min and then 10,000 g at 4 °C for 10 min. The supernatant was collected, which contained cytoplasm and membrane fraction. After centrifuging in an ultracentrifuge at 100,000 g at 4 °C for 1 h, the resulted supernatant was the cytosolic fraction and the pellet would be used for isolating membrane fraction. After resuspending in 1 ml sucrose buffer and centrifuging at 100,000 g at 4 °C for 30 min, the pellet was resuspended with or without 100 µl Na_2_CO_3_ (50 mM) and incubated for 5 min. Then it was resuspended again with 1 ml sucrose buffer and centrifuged at 100,000 g 4 °C for 30 min. The resulting pellet was the membrane fraction, which was dissolved in 100 µl NL buffer (50 mM Tris, 150 mM NaCl, 1% NP-40, 0.5% sodium deoxycholate, 0.1% SDS,1 × protein inhibitor and phosphatase inhibitor, pH 8.0) for detection.

The cytoplasm fraction and nuclear fraction was separated by PARIS™ Kit (Cat# AM1921, Invitrogen) following the manufacturer’s instructions. Briefly, about 200,000 cells were trypsinized and washed in PBS gently. After removing PBS, cells were resuspended in 300 μl ice-cold Cell Fractionation Buffer and incubated on ice for 10 min. A centrifuge at 500 × g 4 °C for 5 min was performed and the supernatant was cytoplasm fraction. The nuclear pellet was then washed with 300 μl ice-cold Cell Fractionation Buffer and centrifuged again. The washed nuclear pellet was now resuspended in 300 μl ice-cold Cell Disruption Buffer and vortexed vigorously to lyse the nuclei.

### Cell surface biotinylation assay

For cell surface biotinylation, cells were cultured in 10 cm dish until 90% confluency. Cells were rinsed twice with ice-cold 1 × PBS and then placed on ice. Next, the membrane impermeable sulfo-NHS-SS-biotin reagent (Cat# PG82077, Thermo Scientific) was added at the final concentrate at 0.5 mM. Cells were then incubated in the dark for 30 min, followed by washing with ice-cold PBS. Then cells were lysed in IP buffer on ice for 30 min and centrifuged at 12,000 rpm for 20 min. The collected supernatant was incubated with Streptavidin-agarose beads (Cat# 20,347, Thermo Fisher Scientific) at 4 °C for 7 h. The immunoprecipitated proteins were then collected and subjected to immunoblotting to examine the proteins in cell membrane fraction.

### Immunohistochemistry (IHC)

IHC was performed on FFPE tissues from iCCA patients. RRM2 antibody (Cat# ab57653, Abcam) and 2-step plus® Poly-HRP Anti-Mouse/Rabbit IgG EnVision Detection System (PV-8000, ZSGB-BIO, China) was used. For each sample, the staining area was evaluated from 1 to 4 (1, 0–25%; 2, 25–50%; 3, 50–75%; 4, > 75%) and the intensities were graded from 0 to 3 (0, negative; 1, weak; 2, moderate; 3, strong). A final IHC score between 0 and 12 was achieved by multiplication of staining area and intensity. For some cases, the staining of RRM2 cell membrane localization or RRM2 cytoplasm localization showed heterogeneity in different areas of tumor tissues. In this case, the final staining score were the summed results from the scores of the different heterogenous areas (mainly 1–3 areas). In addition, the membrane staining score and the cytoplasmic staining score were also calculated separately, based on the same quantifying methods as above.

### Immunofluorescence (IF)

Cells were seeded on coverslips, and then fixed with 4% Paraformaldehyde for 20 min. After being permeabilized with 0.1% Triton X-100 for 10 min, or without permeabilization, the coverslips were blocked with 3% BSA for 30 min and incubated with primary antibodies at 4 °C for overnight. The cells were then incubated with the corresponding secondary antibodies for 1 h and nuclei were stained with DAPI in the mounting reagent (Cat# E607303, Sangon Biotech). Confocal fluorescence images were captured using Zeiss LSM 880/900 AiryScan laser microscope. These antibodies were anti-RRM2 (Cat# ab57653, Abcam), anti-RRM2(Cat# DF-7248, Affinity), anti-Flag Mouse Monoclonal Antibody (Cat# F3165, Sigma), anti-Flag Rabbit Monoclonal Antibody (Cat# AE092, Abclonal), Alexa Fluor ® 488 goat anti-mouse IgG (H + L) (Cat# ab150113, AbCAM) and Anti-Rabbit lgG(H + L), F(ab’)2 Fragment (Alexa Fluor ® 594 Conjugate) (Cat# 8889S, CST). For EdU labeling assay, the BeyoClick^TM^EdU-488 kit was used (Cat# C0071L, Beyotime). Briefly, the sorted RRM2^+^ and RRM2^−^ iCCA cells were treated with 10 μM EdU for 2 h firstly and then fixed with 4% Paraformaldehyde for 20 min. After being permeabilized with 0.1% Triton X-100 for 10 min, cells were covered by Click Additive Solution buffer for 30 min and stained with DAPI in the mounting reagent.

### RNA extraction, quantitative real-time PCR and RNA-sequencing

Total RNA was extracted using TRIzol RNA isolation Reagents (Invitrogen) following the manufacturer’s instructions. cDNA was reverse transcribed with 1 μg of total RNA using PrimeScriptTM RT reagent Kit (Cat# RR047, TaKaRa). Quantitative reverse transcription polymerase chain reaction (qRT-PCR) was performed with the TB Green Premix Ex Taq II (Cat#RR420, TaKaRa). 18S was used as reference gene. All primer sequences are listed in Table S1.

For RNA-sequencing, total RNA of the sorted RRM2^+^ and RRM2^−^ HUCCT1 cells were extracted using TRIzol RNA isolation Reagents. RNAs with a R260/280 2.0 were send to Hangzhou Lianchuan Biotechnology Corporation (Hangzhou, China) for the cDNA library construction and RNA sequencing. Two paired samples were used. The classification criteria for differentially expressed genes (DEGs) were fold change (FC) ≥ 1.2 (RRM2^+^ vs. RRM2^−^ or RRM2^−^ vs. RRM2^+^), FPKM > the third quartile value. 121 genes were identified as the DEGs between RRM2^+^ and RRM2^−^ cells.

### Signal peptide and transmembrane domain prediction

The following online tools were used for predictions of RRM2 signal peptide and transmembrane domain. Phyre2 (http://www.sbg.bio.ic.ac.uk/phyre2) [29] was used for both signal peptide and transmembrane domain prediction. Signal-CF (http://www.csbio.sjtu.edu.cn/bioinf/Signal-CF/) [30] was used only for signal peptide prediction. Meanwhile, Phobius (https://phobius.sbc.su.se/) [31], TOPCONS (https://single.topcons.net) [32], Psipied (http://bioinf.cs.ucl.ac.uk/psipred/) [33] and DNAMAN 6.0.3.99 were used for protein transmembrane domain prediction.

### Statistical analysis

Hierarchical clustering was performed by the GENESIS version 1.7.7 developed by Alexander Sturn (IBMT-TUG, Graz, Austria). Gene set enrichment analysis (GSEA) in the Molecular Signatures Database was performed using GSEA V4.2.2. Kaplan–Meier survival analysis was used to compare patient survival and mouse survival among different groups using GraphPad Prism V8.0 (San Diego, CA), and the *P*-value was generated by the Log-Rank test. Two-way ANOVA and student’s t-test were used for statistical analysis of comparative data between groups. All *P*-values were 2-sided, and *P*-value should be less than 0.05 as significant difference.

## Results

### RRM2 was highly expressed in a stem cell-like malignant population of iCCA

**Fig. 1:**
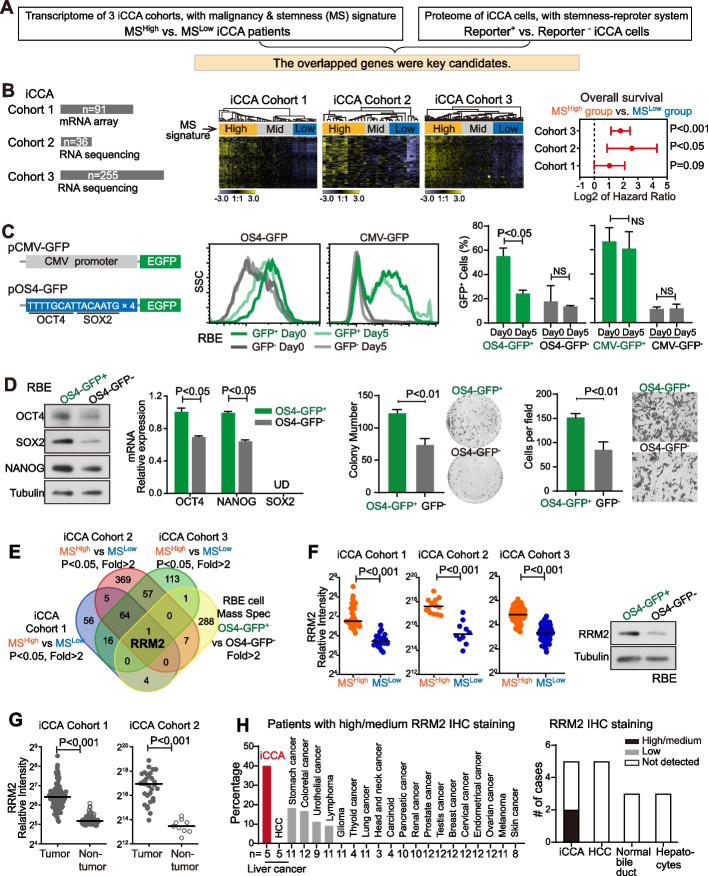
RRM2 was highly expressed in a stem cell-like malignant population of iCCA. **A** Screening strategy to identify the key candidates which were highly expressed in iCCA subpopulations with malignant and stemness (MS) features. **B** The information of iCCA cohorts 1–3 and the hierarchical clustering analysis based on the activation status of the MS signature. iCCA patients were divided into MS^High^, MS^Mid^ and MS^Low^ groups. Samples and genes are displayed as columns and rows, respectively. Hazard ratio of overall survival of MS^High^ group vs. MS^Low^ group were shown and log-rank test was performed. **C** The construction of OS4-GFP reporter was shown in the left. The GFP fluorescence level of GFP^+^ RBE and GFP^−^ RBE cells was analyzed by flow cytometry. Percentage of GFP^+^ cells at day 0 and at the day 5 after cell sorting was quantified. **D** OCT4, NANOG and SOX2 levels in OS4-GFP^+^ RBE cells and OS4-GFP^−^ RBE cells. Colony formation and cell migration were performed and compared between OS4-GFP^+^ RBE cells and OS4-GFP^−^ RBE cells. **E** Venn diagram of molecules with significantly higher expression in MS^high^ group vs. MS^low^ group in each cohort and in OS4-GFP^+^ vs. OS4-GFP^−^ RBE cells (fold > 2). **F** Relative expression level of RRM2 in MS^High^ group and MS^Low^ group of iCCA cohorts 1–3 and RRM2 protein level in OS4-GFP^+^ RBE cells and OS4-GFP^−^ RBE cells. **G** Relative expression level of RRM2 in iCCA tumor tissues and non-tumor tissues in iCCA cohorts 1–2. **H** Percentage of high/medium IHC staining of RRM2 in 21 types of cancers and number of cases with different RRM2 IHC staining in iCCA tumors, normal bile ducts and hepatocytes. The information was collected from the Human Protein Atlas. (C, D, F, G) The Student’s *t*-test was used

To identify and characterize iCCA subpopulations with severe malignant features, an unbiased global screening strategy was established (Fig. 1A). A well-recognized malignancy/stemness (MS) signature including 84 genes was used to classify iCCA patients with MS signature [34–37]. A stemness-reporter system was established to sort iCCA cells with malignant and stemness features. With the identified malignant iCCA patients and iCCA cells, the MS related genes were explored.

The effectiveness of MS signature was established in breast cancer and then utilized in several different cancer types such as retinoblastoma and gastric cancer [34–37]. Here we firstly evaluated this signature in HCC, the most common liver cancer, as its malignancy and stemness features were well studied. In two HCC cohorts (Table 1), hierarchical clustering analysis with the MS signature revealed distinct groups with different activation levels of the MS signature. The MS-high activation (MS^high^) group exhibited a significantly worse prognosis (Fig S1A) and a significantly higher expression of several known HCC CSC biomarkers such as EpCAM and CD24 (Fig S1B). These indicated that the MS signature allowed to identify cancer patients with high malignancy and stemness features. Next, we employed the MS signature to categorize iCCA patients. In iCCA cohorts 1–3 (Table 1), the hierarchical clustering analysis with the MS signature consistently classified iCCA patients to subgroups with different expression levels of MS signature genes. Compared to the corresponding MS^low^ subgroup, iCCA patients in the MS^high^ subgroup had a worse prognosis in all three iCCA cohorts (Table S2-4, Fig. 1B) and the statistical significance was reached in iCCA cohorts 2 and 3. Meanwhile, the MS^high^ subgroup also contained more iCCAs with a higher rate of intrahepatic metastasis and vascular invasion (iCCA cohort 3, *P* < 0.01, Table S4).

Oct4 and Sox2 are known essential transcription factors for pluripotency and self-renewal, as well as cell malignancy features [38–42]. In this study, a GFP reporter with four concatenated repeats of Oct4/Sox2 binding elements in the promoter region was constructed, i.e., OS4-GFP, as the stemness reporter (Fig. 1C). Such a reporter allows the identification of cells with malignancy and stemness features. Consistently in Huh7, an HCC cell line that was previously used for CSC studies [43, 44], OS4-GFP^+^ cells and OS4-GFP^−^ cells were successfully sorted and the sorted OS4-GFP^+^ cells demonstrated malignancy and stemness features (Fig S1C-D). The CMV-GFP vector was used as a negative control. In this case, a OS4-GFP RBE iCCA cell line was established, the OS4-GFP^+^ RBE cells and the OS4-GFP^−^ RBE cells were sorted (Fig. 1C). Five days after culturing, the sorted OS4-GFP^+^ RBE cells yielded to a mixed OS4-GFP^+^ and OS4-GFP^−^ population, whereas the sorted OS4-GFP^−^ fraction did not produce OS4-GFP^+^ cells (Fig. 1C). As a negative control, CMV-GFP^+^ RBE cells only showed minimal changes after 5 days culture. Moreover, the expression of stemness-related genes OCT4, SOX2 and NANOG was noticeably higher in OS4-GFP^+^ RBE cells compared to OS4-GFP^−^ RBE cells (Fig. 1D). OS4-GFP^+^ RBE cells also possessed significantly stronger abilities of colony formation and migration ability than OS4-GFP^−^ RBE cells (*P* < 0.05) (Fig. 1D). Consistently, OS4-GFP HUCCT1 iCCA cell line was also established and the sorted OS4-GFP^+^ HUCCT1 cells demonstrated the comparable phenotype (Fig S1E-G). Moreover, OS4-GFP^+^ HUCCT1 cells also possessed significantly stronger spheroid formation ability than OS4-GFP^−^ iCCA cells (*P* < 0.05) (Fig S1F). These results demonstrated that OS4-GFP^+^ iCCA cells were a malignant cell subpopulation with CSC-like properties.

Next, genes with a significantly higher level in these malignant iCCA patients and iCCA cells were explored via comparing tumor transcriptomic profiles of MS^high^ patients vs. MS^low^ patients in three iCCA cohorts and proteomic profiles of OS4-GFP^+^ vs. OS4-GFP^−^ RBE cells. Molecules with significant two-fold higher expression in MS^high^ group vs. MS^low^ group in each iCCA cohort and in OS4-GFP^+^ RBE vs. OS4-GFP^−^ RBE cells were chosen as key candidates (Fig S2A-D). Venn diagram analysis of four comparisons revealed RRM2 as the top candidate (Fig. 1E). As shown in Fig. 1F, RRM2 mRNA level was significantly higher in the MS^high^ group than in the MS^low^ group in iCCA cohorts 1–3 (*P* < 0.001). The RRM2 protein level was much higher in OS4-GFP^+^ cells than in OS4-GFP^−^ cells sorted from either RBE cell line (Fig. 1F) or HUCCT1 cell line (Fig S1G). Moreover, RRM2 expression level was significantly increased in iCCA tumor tissues compared to non-tumor tissues (iCCA cohorts 1–2, *P* < 0.001, Fig. 1G). The IHC staining results from the Human Protein Atlas (HPA) suggested that there was the highest percentage of high/medium staining of RRM2 in iCCAs among 21 different types of cancers, while no RRM2 staining was detected in normal bile duct cells or hepatocytes (Fig. 1H).

### Silencing RRM2 significantly suppressed the malignancy and stemness features of iCCA cells

**Fig. 2:**
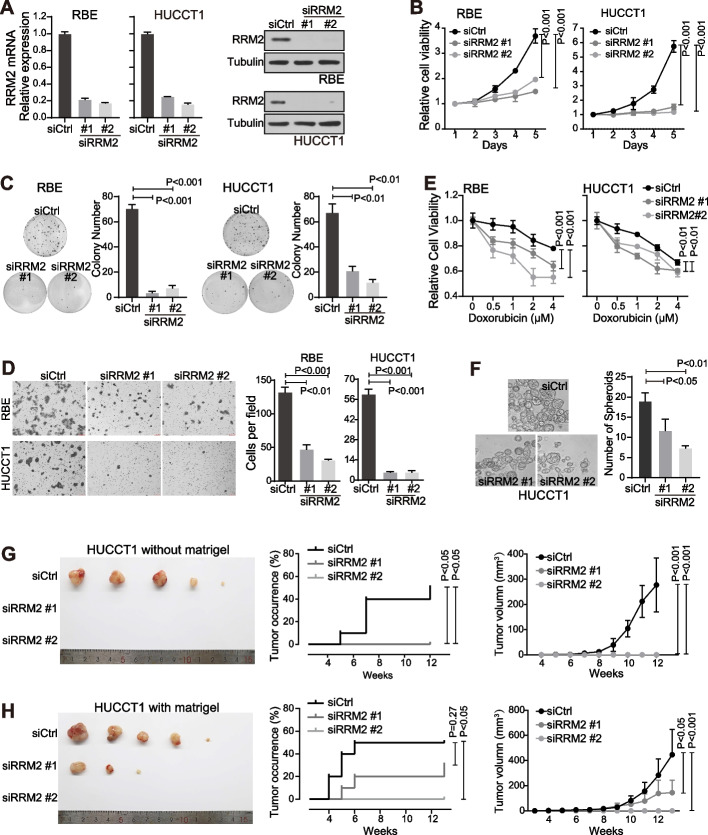
Silencing RRM2 significantly suppressed malignancy and stemness features of iCCA cells. **A** Silencing efficiency of RRM2 siRNAs in RBE and HUCCT1 cells was evaluated by qRT-PCR and western blot. **B**-**D** Cell viability (B), colony formation (C) and cell migration (D) were performed in RBE and HUCCT1 cells transfected with siCtrl or siRRM2. **E** Cell viability was examined in RBE and HUCCT1 cells transfected with siCtrl or siRRM2 followed by Doxorubicin treatment. **F** Spheroid formation assay was performed in HUCCT1 cells transfected with siCtrl or siRRM2. Spheroids with diameter ≥ 50 μm were counted. **G, H** Tumorigenicity assay with 1 × 10^4^ HUCCT1 cells transfected with siCtrl and siRRM2 with or without Matrigel in male BALB/c nude mice. Five mice for each group (siCtrl, siRRM2#1 and siRRM2#2) were used. Images of tumors derived from nude mice were shown. Tumor occurrence rate and tumor volume were compared. **B**, **E**, **G**, **H** Two-way ANOVA was used. **C**, **D**, **F** The Student’s *t*-test was used

RRM2 was reported to increase malignancy features of several cancers, but not yet in iCCA. Therefore, RRM2 silencing using siRNAs was performed in iCCA cells to evaluate its role in promoting iCCA malignancy (Fig. 2A). RRM2 siRNAs significantly reduced the mRNA and protein levels of RRM2 in both RBE and HUCCT1 iCCA cells. Consistently, RRM2 silencing significantly suppressed cell malignancy features such as cell proliferation (Fig. 2B), colony formation (Fig. 2C) and cell migration (Fig. 2D) in both iCCA cell lines. Moreover, RRM2 silencing also reduced the stemness features of iCCA cells. As shown in Fig. 2E, RRM2 silencing increased the sensitivity of both RBE and HUCCT1 cells to the treatment of doxorubicin, a commonly used chemotherapeutic drug. RRM2 silencing in HUCCT1 cells also significantly reduced the spheroid formation (Fig. 2F), which represented the self-renewal ability of cells.

Consistent data were also obtained in vivo. HUCCT1 cells with or without RRM2 silencing were used for the tumorigenicity assay. As shown in Fig. 2G-H, RRM2 silencing significantly delayed the tumor onset time, and decreased the rate of tumor occurrence as well as tumor size. RBE cells could not form spheroids in vitro or initiate tumors in BALB/c nude mice in vivo (even with Matrigel, Data not shown). Together, these findings strongly indicated that RRM2 silencing significantly suppressed iCCA malignancy and stemness features.

### RRM2 presented cell membrane localization in iCCA tumors

**Fig. 3:**
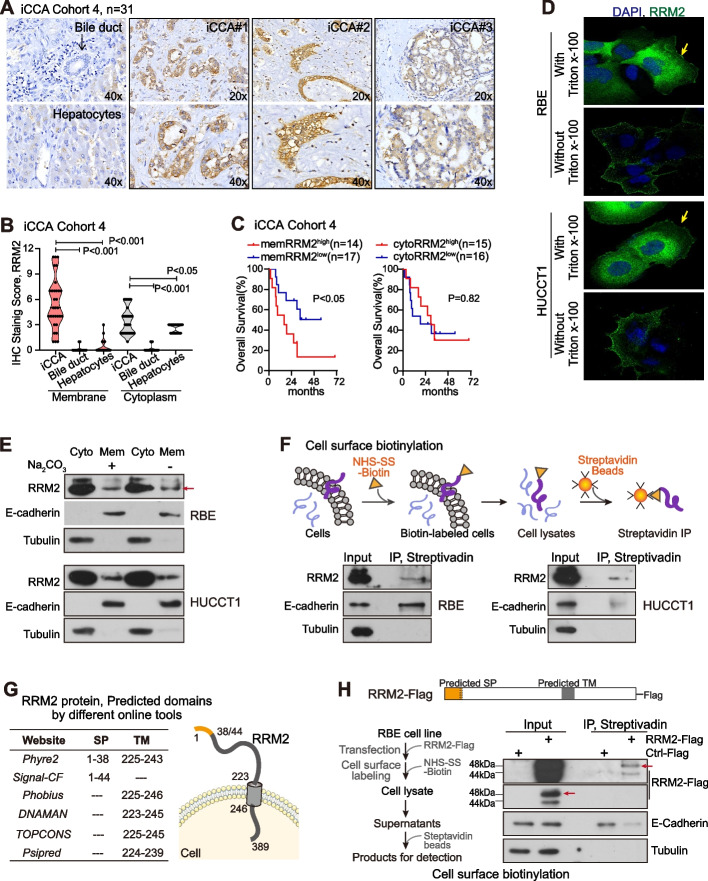
RRM2 presented cell membrane localization in iCCA tumors. **A** Representative images of RRM2 IHC staining in iCCA tumors, normal bile duct and hepatocytes of iCCA cohort 4. **B** IHC staining scores for RRM2 membrane localization and cytoplasm localization were measured in iCCA tumors, normal bile duct and hepatocytes, the Student’s *t*-test was used. **C** Kaplan–Meier analysis of overall survival in cohort 4 based on RRM2 membrane staining scores or cytosol staining scores. Log-rank test was performed. Mem, membrane; Cyto, cytoplasm. **D** Confocal microscopy images of endogenous RRM2 in RBE and HUCCT1 cells with or without permeabilization by Triton X-100. Yellow arrows indicate cell membrane localization of RRM2. **E** Cell fractionation assay by ultra-highspeed centrifugation was performed in RBE and HUCCT1 cells with or without Na_2_CO_3_ treatment. **F** Cell surface biotinylation assay was performed in RBE and HUCCT1 cells. **G** The predicted protein structure of RRM2 according to multiple prediction online tools. SP, signal peptide; TM, transmembrane domain. **H** The diagram depicting RRM2-Flag vector and cell surface biotinylation assay with RBE cells transfected with RRM2-Flag

The expression of RRM2 in iCCA patients was evaluated with immunohistochemistry (IHC) in iCCA cohort 4, which included 31 paired FFPE iCCA tumors and adjacent non-tumor FFPE liver tissues (Table 1). Significantly, RRM2 IHC staining was generally strong in most iCCA tumor tissues, but it was weak in hepatocytes and no staining in bile duct cells or other cells in the tumor microenvironment (Fig. 3A). The quantitative data revealed a statistically significant difference (*P* < 0.001, Fig S3). RRM2 was reported to mainly locate in the cytoplasm and occasionally in the nucleus, which was also noticed in iCCA tumor tissues. In addition, our results revealed that RRM2 also presented an extensive cell membrane localization in the majority of iCCA tumor tissues (Fig. 3A), which has not been reported before.

We then analyzed the association of the different localizations of RRM2 protein in iCCA cells with the clinical relevance of iCCA patients (Table S5). The cytoplasm staining and the cell membrane staining of RRM2 were quantified respectively. The quantitative data revealed that both RRM2 cell membrane staining and its cytoplasm staining were significantly higher in iCCA tumor cells compared to the corresponding bile duct cells and hepatocytes (P < 0.05 for each comparison, Fig. 3B). The cell membrane staining of RRM2 in iCCA tumor tissues was even stronger than its cytoplasm staining. Moreover, iCCA patients with higher RRM2 staining on the cell membrane (score ≥ 6, median cut-off) had worse prognosis than patients with low RRM2 cell membrane staining (score ≤ 5), whereas the cytoplasmic localization of RRM2 was not related to patient prognosis (Fig. 3C).

Next, we thoroughly investigated and confirmed the cell membrane localization of RRM2 via a series of assays. In iCCA cell lines, RRM2 immunofluorescence (IF) was performed under conditions with or without cell membrane permeabilization by triton X-100. In two iCCA cell lines with permeabilization, RRM2 showed a noticeable localization of both cytoplasm and membrane. When permeabilization was not applied, RRM2 only exhibited a sharp clear cell membrane localization. In this IF assay, the RRM2 antibody recognizing its N-terminus was used, indicating an extracellular localization of RRM2 N-terminus (Fig. 3D). Cell fractionation assay via ultracentrifuge was also carried out to extract the cytoplasm and membrane fraction separately. Notably, RRM2 presented in both fractions in two iCCA cell lines (Fig. 3E). Further, Na_2_CO_3_ was used in this assay since that Na_2_CO_3_ was known to remove attached peripheral membrane protein from the membrane fraction but not disrupt the integrity of the membrane. Significantly, upon the Na_2_CO_3_ exposure, RRM2 protein remained in the membrane fraction, suggesting the potential transmembrane localization of RRM2 (Fig. 3E). Furthermore, cell surface biotinylation assay was performed with biotin labelling and streptavidin-IP, as illustrated in Fig. 3F. Sulfo-NHS-SS-Biotin is negatively charged and does not permeate the cell membrane so that it labels only extracellular peptides of membrane proteins. Thus, the input samples were whole cell lysates while the IP products were cell membrane fraction. Consistently, RRM2 was detected in the IP samples, further supporting the membrane localization of RRM2 and the existence of extracellular domain of RRM2 (Fig. 3F). Together, these results demonstrated the cell membrane localization of RRM2 in iCCA and that its membrane localization was related to poor prognosis of iCCA patients.

### RRM2 possessed an unclassical signal peptide (1-44aa), essential for RRM2 cell membrane localization

With several widely used online tools, RRM2 protein sequence was predicted to contain a signal peptide (SP, 1-38aa or 1-44aa) and a transmembrane domain (223-246aa), and present its N-terminal region in the extracellular space (Fig. 3G). According to the prediction, RRM2-Flag vector was constructed with a Flag tag at RRM2’s C-terminus. Cell surface biotinylation assay was then performed using the RRM2-Flag construct. Consistently, the exogenous RRM2 could be also detected in the membrane fraction (Fig. 3H).

**Fig. 4:**
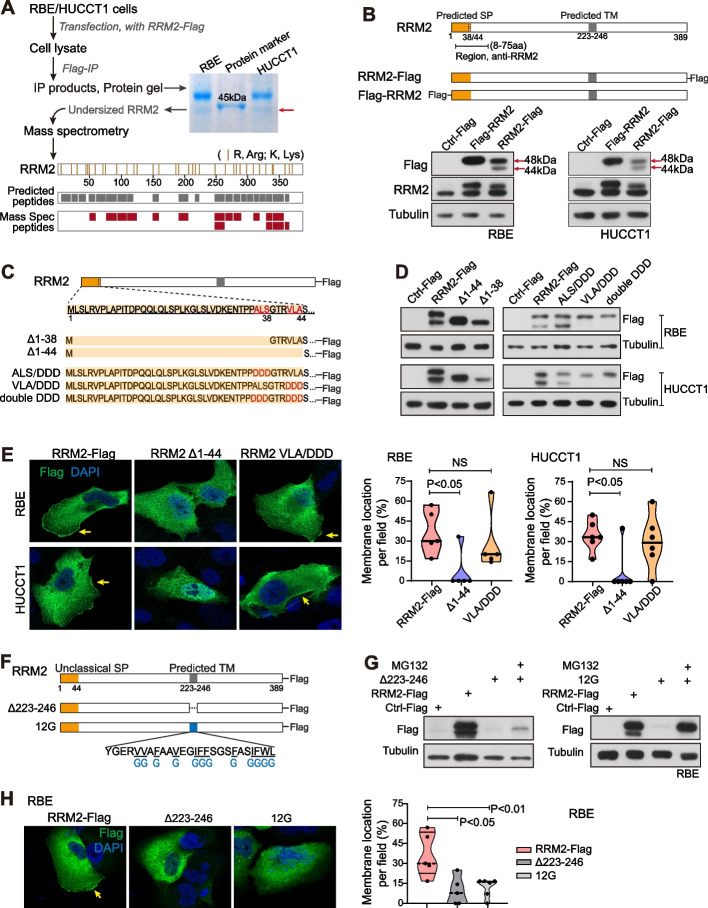
The unclassical signal peptide and the potential transmembrane domain were examined and essential for RRM2 cell membrane localization. **A** The flow chart of IP/Mass Spec to assess the undersized RRM2. The predicted peptides (up, grey) and Mass Spec detected peptides (down, dark red) of RRM2 were shown. Yellow lines represent “K” or “R” residues of RRM2. **B** Construction of RRM2 vectors, and the expression of Flag-RRM2 and RRM2-Flag in RBE and HUCCT1 cells. **C** Schematic diagram of a group of RRM2 truncations and mutations related to its signal peptide. **D** The expression of RRM2 with different truncations and mutations of SP region was detected by western blot using anti-Flag. **E** Confocal microscopy images of exogenous RRM2 detected by anti-Flag in RBE and HUCCT1 cells. Yellow arrows indicate the cell membrane localization of RRM2. Percentage of RRM2 cell membrane localization was measured (mean ± SD). **F** Schematic diagram of RRM2 vectors with truncation and mutation of the predicted TM region. **G** The expression of RRM2 with 223-246aa truncation or mutations of hydrophobic amino acids in 223-246aa region was detected by anti-Flag in RBE cells. **H** Confocal microscopy images of exogenous RRM2 detected by anti-Flag in RBE cells. Yellow arrows indicate the cell membrane localization of RRM2. Percentage of RRM2 cell membrane localization was measured (mean ± SD). (E, H) The Student’s *t*-test was used

Notably, when RRM2-Flag was overexpressed in two iCCA cell lines, two protein bands (48 kDa and 44 kDa) were noticed in both whole cell lysates and cell membrane fraction. The 48 kDa was the calculated size for RRM2-Flag. There was no additional translational start codon after the first ATG codon in the RRM2-Flag vector. The 44 kDa band did not seem to be produced from the proteasomal or lysosomal degradation of RRM2 either (Fig S4A). Meanwhile, the predicted SP of RRM2 was around 40aa, contributing ~ 4 kDa to the protein size. Thus, the 44 kDa RRM2 might be a potentially cleaved form of RRM2 without the predicted SP. To test this possibility, we performed IP/Mass Spec analysis of the 44 kDa RRM2-Flag (Fig. 4A). In the process of Mass Spec, Trypsin was used to digest proteins at lysine (K) and arginine (R) residues. Via comparing the predicted peptides (7 ~ 25aa) generated by trypsin digestion and the detected peptides via Mass Spec, we noticed that there were no peptides detected in the 1-50aa region of RRM2’s N-terminus. This result was in parallel with the hypothesis that the 44 kDa RRM2 was the cleaved form without the predicted SP.

As the SP is located at the protein’s N-terminus, placing a tag at the N-terminus of RRM2 would destroy such an SP cleavage. Consistently, Flag-RRM2 was constructed with a Flag tag at RRM2’s N-terminus and immunoblotting results showed only a single 48 kDa band of Flag-RRM2 in iCCA cells (Fig. 4B). In the literature, the vector of RRM2 with tag at its N-terminus was used for the functional assays [17, 27, 45], but RRM2-Flag (RRM2 vector with Flag tag at its C-terminus) was not. Meanwhile, RRM2 was reported to be mainly localized in the cytoplasm [17, 46] and such a location was also noticed in iCCA (Fig. 3A). In this case, the predicted SP of RRM2 was likely an unclassical signal peptide and only functioned under certain circumstances.

The SP region of RRM2 was then determined. It is known that SP contains a signal peptidase cleavage site at the end of the SP C-terminus. Such a site includes three amino acids, among which the 1st and 3rd amino acids have to be small and neutral for cleavage to occur correctly [47]. Two regions of RRM2, i.e., 1-38aa, 1-44aa, were predicted as the potential SPs. To investigate which of them was the RRM2 SP, we generated two RRM2 truncations (1-38aa truncation, Δ1-38; 1-44aa truncation, Δ1-44) and two RRM2 mutations with the possible SP cleavage sites (Ala-Leu-Ser, ALS, 36-38aa; Val-Leu-Ala, VLA, 42-44aa) being mutated to the negatively charged triple Asp (DDD) (Fig. 4C). In both iCCA cell lines, either of truncation vectors expressed one single band with a comparable size to the short 44 kDa RRM2-Flag (Fig. 4D). Meanwhile, the 44 kDa RRM2 disappeared when VLA at the 42-44aa was mutated to DDD (VLA/DDD), but still remained when ALS at the 36-38aa was mutated to DDD (ALS/DDD) (Fig. 4D). Moreover, the consistent data were also obtained when VLA (42-44aa) or ALS (36-38aa) of RRM2 was deleted, i.e., the 44 kDa RRM2 disappeared when VLA was deleted, but remained when ALS was deleted (Fig S4B). Together, these data demonstrated that VLA at the 42-44aa is the recognition site of signal peptidase and the 1-44aa region is the unclassical SP of RRM2.

Furthermore, roles of the unclassical SP in RRM2 cell membrane trafficking were investigated. In iCCA cells, RRM2-Flag, RRM2 Δ1-44 and RRM2 VLA/DDD vectors were transfected and IF assay was performed. As shown in Fig. 4E, RRM2-Flag showed both cytoplasm and cell membrane localization. However, when the SP region of RRM2 was removed, RRM2 cell membrane localization was significantly reduced in comparison to RRM2-Flag (*P* < 0.05, Fig. 4E). Meanwhile, there was no significant difference between the localizations of RRM2 VLA/DDD and RRM2-Flag. These data suggested that the presence of the SP was required for RRM2’s cell membrane localization, whereas the SP cleavage was not necessary. This result was consistent with the results in Fig. 3H that both the un-cleaved and cleaved forms of RRM2 (48 kDa and 44 kDa) could be detected at the cell membrane fraction.

### RRM2 possessed a potential transmembrane domain, essential for RRM2 cell membrane localization

According to the sequence analysis of RRM2, we further investigated the 223-246aa region of RRM2, which is the predicted transmembrane domain. Two RRM2 vectors were constructed (Fig. 4F), i.e., one with the 223-246aa deletion (Δ223-246), the other with all 12 hydrophobic amino acids in the 223-246aa region being mutated to Gly (12G). As shown in Fig. 4G, both vectors had low expression efficiency in RBE cells while the treatment with MG132, a proteasome inhibitor, rescued the RRM2 expression level. Upon MG132 treatment, IF showed that the cell membrane localization of RRM2 was significantly reduced when 223-246aa was deleted (*P* < 0.05) or its hydrophobic amino acids were mutated (*P* < 0.01) in RBE cells (Fig. 4H). Consistent results were obtained in the other iCCA cell line HUCCT1 (Fig S4C). In addition, comparable data were also obtained in iCCA cells without MG132 exposure (Data not shown). These results suggested that the 223-246aa region was a potential transmembrane domain for RRM2, essential for the cell membrane localization of RRM2. In addition, this region was also important for RRM2’s protein stability, which was interesting for a future study.

**Fig. 5:**
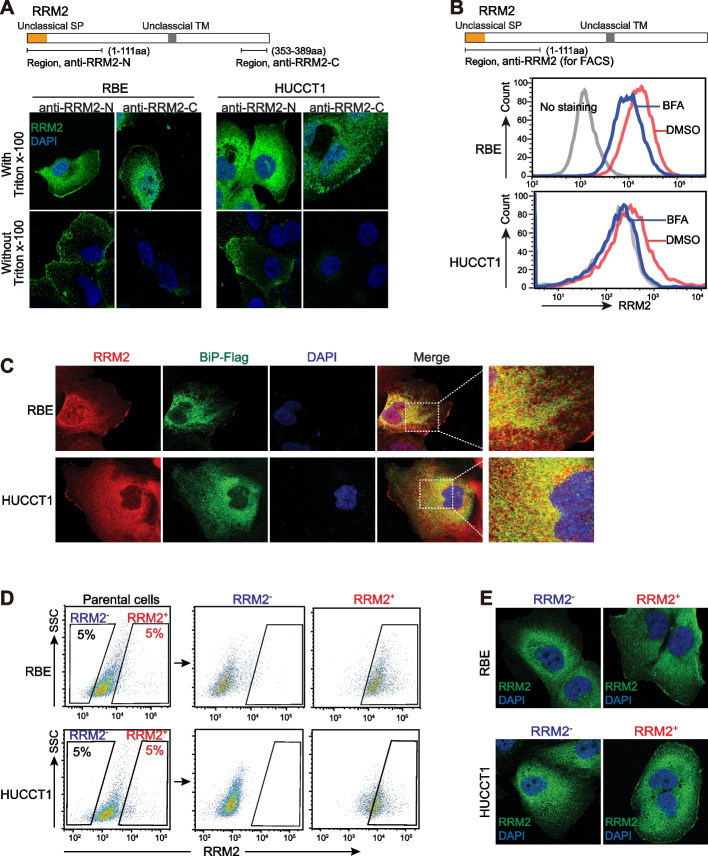
RRM2 trafficked to cell membrane via classical ER-Golgi pathway and membrane RRM2 positive cells could be enriched via cell sorting. **A** Confocal microscopy images of endogenous RRM2 in RBE and HUCCT1 cells with or without permeabilization detected by a RRM2 N-terminus antibody and a RRM2 C-terminus antibody. **B** Membrane RRM2 staining in RBE and HUCCT1 cells was determined by flow cytometry via using the antibody which recognized 1-111aa of RRM2, with or without BFA treatment. Grey line, no staining; red line, DSMO treatment; blue line, BFA treatment. **C** Confocal microscopy images of endogenous RRM2 and BiP and their co-localization in RBE and HUCCT1 cells. **D** Flow cytometry analysis of parental iCCA cells and the sorted membrane RRM2^+^ cells (the top 5% of cells from the RRM2 staining) and the sorted membrane RRM2^−^ cells from parental RBE and HUCCT1 cells. The antibody recognizing 1-111aa of RRM2 was used. **E** IF assay was performed with RRM2 antibody for the sorted RRM2^+^ and RRM2^−^ cells at day 1 after cell soring. Confocal microscopy images of endogenous RRM2 were shown

When examining the endogenous RRM2 localization in iCCA tissue and iCCA cells, a RRM2 antibody recognizing its N-terminal 1-111aa was used and cell membrane localization of RRM2 was noticed (Fig. 3). In the IF assay without cell permeabilization, this antibody still detected the cell membrane localization of RRM2 (Fig. 3D, and Fig. 5A), indicating an extracellular localization of RRM2 N-terminus, in parallel with the prediction of RRM2 sequence analysis (Fig. 3G). Comparably, when a RRM2 antibody recognizing its C-terminal 353-389aa was used, RRM2 showed cell membrane localization only when cell permeabilization was applied (Fig. 5A). Thus, the N terminal region of RRM2 was extracellular when RRM2 presented as a cell membrane protein.

### RRM2 trafficked to the cell membrane via classical ER-Golgi pathway and membrane RRM2 positive cells could be enriched via cell sorting

Membrane proteins with SP are usually trafficking to the cell membrane through the classical ER-Golgi secretory pathway. We then tested whether RRM2’s membrane trafficking was also through this classical way. Brefeldin A (BFA), an ER-Golgi trafficking inhibitor was then used and the flow cytometry was performed with the RRM2 N-terminus antibody. As shown in Fig. 5B, the RRM2 positive population was detected in both RBE and HUCCT1 cells, which was remarkedly reduced by BFA. Consistent data were obtained when golgicide A (GCA), the other ER-Golgi trafficking inhibitor, was used (Fig S5A). Moreover, the transient localization of RRM2 in the ER and Golgi was analyzed with the ER residential protein BiP as an ER marker and the Golgi residential membrane protein GOLPH2 as a Golgi marker. The results showed that BiP and GOLPH2 were mainly located around the nuclear region while RRM2 was widely distributed including nucleus, cytoplasm and cell membrane. The co-localization revealed that RRM2 partially co-localized with BiP in the ER (Fig. 5C) and with GOLPH2 in the Golgi (Fig S5B) around the nucleus. Together, these data indicated that RRM2 could traffic to cell membrane via the classical ER-Golgi pathway.

According to the results above, it was likely that membrane RRM2-positive iCCA cells could be enriched via cell sorting with the RRM2 N-terminus antibody. Consistently, cell sorting was performed with the top 5% of RRM2 positive cells as RRM2^+^ iCCA cells, and the corresponding 5% of least positive cells as RRM2^−^ iCCA cells (Fig. 5D). Their RRM2 membrane staining was confirmed by IF assay (Fig. 5E). The sorted RRM2^+^ cells possessed a clear cell membrane staining of RRM2, while the corresponding RRM2^−^ cells did not. In both populations, cytoplasm RRM2 staining was positive. These data were consistent in both RBE and HUCCT1 iCCA cells.

### Membrane RRM2-positive cells represented a malignant population with CSC features

**Fig. 6:**
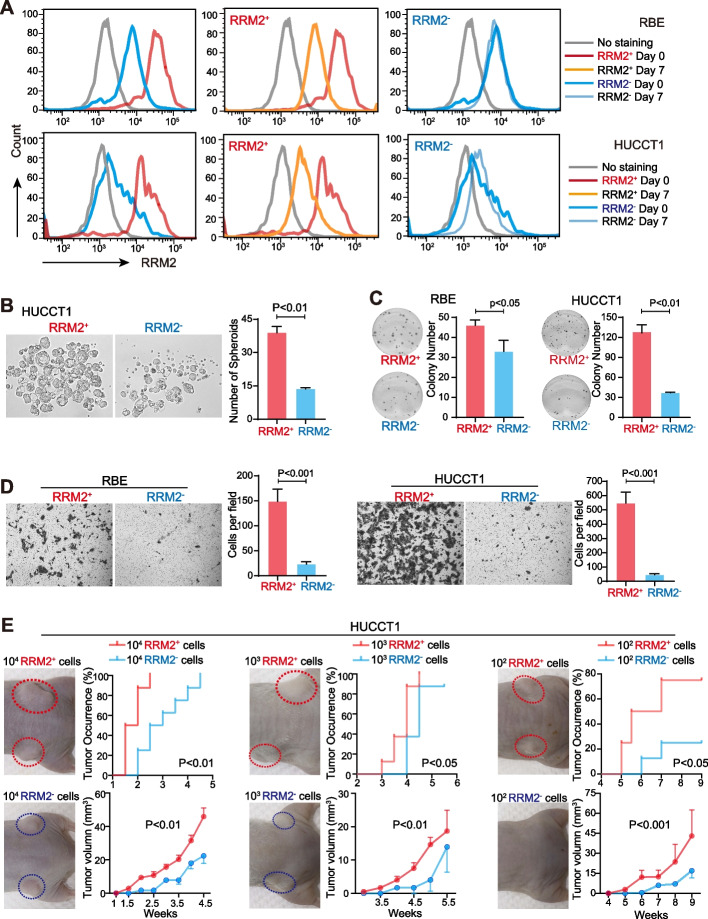
Membrane RRM2^+^ cells were a malignant population with CSC phenotypic features. **A** RRM2^+^ cells and RRM2^−^ cells were sorted by FACS and analyzed by flow cytometry in RBE and HUCCT1 cells at day 0 and day 7 after cell sorting. **B** Spheroid formation assay in RRM2^+^ HUCCT1 and RRM2^−^ HUCCT1 cells. Spheroids with diameter ≥ 50 μm were counted. **C, D** Colony formation and cell migration assays in RRM2^+^ and RRM2^−^ iCCA cells. **E** Tumorigenicity assay was performed with RRM2^+^ HUCCT1 and RRM2^−^ HUCCT1 cells in male BALB/c nude mice. Four mice for each group were used. Tumor occurrence rate and tumor volume were compared. The representative images were shown. (B-D) The Student’s *t*-test was used. (E) Two-way ANOVA was used

We then investigated malignancy features of the sorted RRM2^+^ iCCA cells. The differentiation ability was foremost evaluated. The sorted RRM2^+^ and RRM2^−^ iCCA cells were cultured. At 7 days after culturing, their membrane RRM2-positive populations were examined and also compared with results from cells before culturing. As shown in Fig. 6A, the membrane RRM2-positive population of RRM2^+^ cells was largely reduced following the culture, whereas such a population in RRM2^−^ cells remained at a low level without changes. Consistent results were obtained in both RBE and HUCCT1 cells. These data indicated that the membrane RRM2-positive iCCA cells underwent differentiation during the culture and gave rise to both membrane RRM2-positive cells and membrane RRM2-negative cells (Fig. 6A).

The spheroid formation assay was performed to determine the cell self-renewal ability. As shown in Fig. 6B, the sorted RRM2^+^ HUCCT1 cells formed a significantly higher number of spheroids compared to the corresponding RRM2^−^ counterparts. This result indicated that membrane RRM2-positive cells possessed stronger self-renewal ability compared to negative cells. Moreover, the membrane RRM2-positive cells also had stronger abilities of colony formation and cell migration than negative cells in both RBE and HUCCT1 cells (Fig. 6C-D). However, MTT assay and 5-ethynyl-2’-deoxyuridine (EdU) labeling revealed that there were no significant differences in cell viability and the percentage of EdU-positive cells between the sorted RRM2^+^ iCCA cells and RRM2^−^ iCCA cells (Fig S6A-B). These results indicated that the sorted RRM2^+^ cells possessed stemness-related features rather than cell proliferation advantage.

Furthermore, tumorigenicity assay in nude mice with limiting dilution was also carried out with the sorted RRM2^+^ and RRM2^−^ HUCCT1 cells. HUCCT1 cells showed a very strong tumor initiation ability as shown in Fig. 2. In this case, 10,000, 1000 and 100 of each fraction were injected subcutaneously into BALB/c nude mice. As shown in Fig. 6E, in each group, membrane RRM2-positive cells formed tumor more effectively and quickly than RRM2^−^ cells, and the tumor size in the RRM2^+^ group was always significantly larger than RRM2^−^ cells. Much more significantly in the group of 100 cells, 100 RRM2^+^ HUCCT1 cells could initiate tumors in 6 out of 8 injected sites, while RRM2^−^ cells only produced 2 tumors among 8 injected sites at 9 weeks after injection (Fig. 6E). Thus, the membrane RRM2-positive iCCA cells were more tumorigenic. Together, these results indicated that membrane RRM2-positive cells were a highly malignant cell population with CSC features.

### Membrane RRM2-positive cells possessed malignant molecular features

**Fig. 7:**
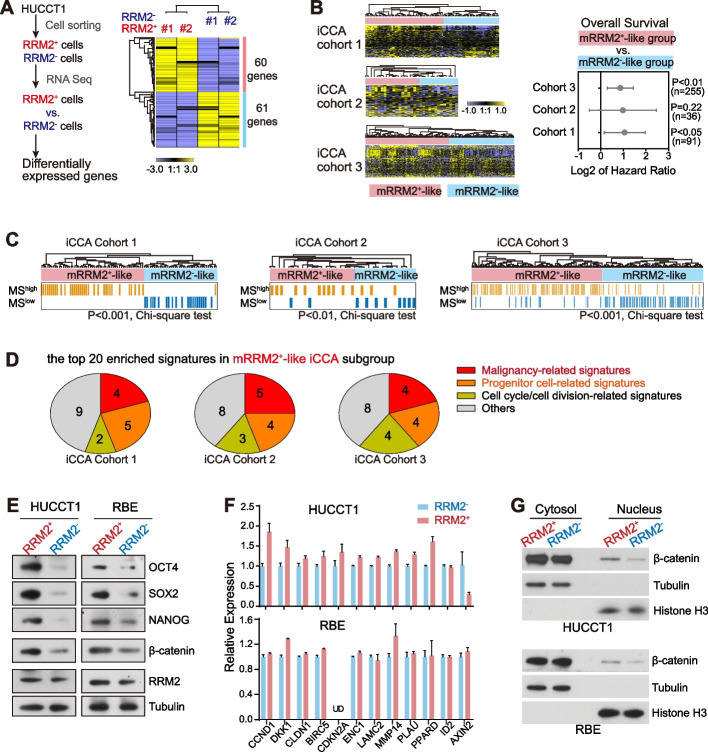
Membrane RRM2^+^ cells possessed the malignant and stemness molecular features. **A** RNA sequencing of the sorted RRM2^+^ HUCCT1 and RRM2^−^ HUCCT1 cells was performed and the differentially expressed genes between two groups were identified. These genes were termed as membrane RRM2 signature. Hierarchical clustering analysis of the sorted RRM2^+^ and RRM2^−^ HUCCT1 cells was performed with this signature. **B** Clustering analysis with the membrane RRM2 signature classified iCCA patients into membrane RRM2-positive (mRRM2^+^)-like group and membrane RRM2-negative (mRRM2^−^) -like group. Hazard ratio of overall survival of mRRM2^+^ -like group and mRRM2^−^ -like group in iCCA cohorts 1–3 was shown. Log-rank test was performed. **C** In iCCA cohorts 1–3, MS signature activation status in mRRM2^+^-like group and mRRM2^−^ -like group. Chi-squared test was performed. **D** The top 20 signatures enriched in mRRM2^+^-like group analyzed by GSEA in iCCA cohorts 1–3. The number of different types of signatures was shown. **E** Protein expression of OCT4, SOX2, NANOG, β-catenin and RRM2 in the sorted RRM2^+^ and RRM2^−^ iCCA cells. **F** Expression of β-catenin direct target genes was detected by qRT-PCR in the sorted RRM2^+^ iCCA and RRM2^−^ iCCA cells. **G** β-catenin protein level in cytoplasm fraction and nuclear fraction in the sorted RRM2^+^ and RRM2^−^ iCCA cells

RNA-sequencing of the sorted HUCCT1 RRM2^+^ cells and RRM2^−^ cells was performed. The differentially expressed genes between RRM2^+^ cells and RRM2^−^ cells (n = 121) were identified based on their fold changes ≥ 1.2 in either RRM2^+^ vs. RRM2^−^ comparison (60 genes) or RRM2^−^ vs. RRM2^+^ comparison (61 genes) from two paired sequenced samples. This group of genes was termed membrane-RRM2 signature (Fig. 7A). With this signature, the hierarchical clustering analysis was performed in iCCA cohorts 1–3. Patients in each cohort were classified into two groups, i.e., one enriched with genes highly expressed in membrane RRM2-positive cells (mRRM2^+^-like iCCAs), and the other enriched with genes highly expressed in membrane RRM2-negative cells (mRRM2^−^-like iCCAs). Consistently, mRRM2^+^-like iCCA patients had a worse prognosis than mRRM2^−^-like iCCAs in three cohorts and the statistical significance was reached in iCCA cohort 1 and cohort 3 (Fig. 7B). This result was consistent with the IHC result in cohort 4 that iCCA patients with strong membrane RRM2 IHC staining had worse prognosis (Fig. 3C). Moreover, iCCA patients in the MS^high^ subgroup were enriched in the mRRM2^+^-like iCCA subgroup while patients in the MS^low^ subgroup were in the mRRM2^−^-like subgroup (Fig. 7C, P < 0.01 for each cohort). In addition, gene set enrichment analysis (GSEA) was carried out to explore the associated molecular signatures within patients of the mRRM2^+^-like iCCA subgroup in the three iCCA cohorts, respectively. The top 20 enriched signatures were analyzed. Among them, three types of signatures in each cohort were highly enriched, i.e., malignancy related signatures (n = 4, 5, 4 in cohorts 1–3, respectively), progenitor or stem cell related signatures (n = 5, 4, 4 in cohorts 1–3, respectively) and cell cycle/cell division related signatures (n = 2, 3, 4 in cohorts 1–3, respectively) (Fig. 7D, Fig S7).

Next, we investigated the expression of stemness-related factors in the sorted RRM2^+^ cells and RRM2^−^ cells. Western blot results revealed that OCT4, SOX2 and NANOG protein levels were noticeably higher in the sorted RRM2^+^ iCCA cells than RRM2^−^ iCCA cells (Fig. 7E). Wnt/β-catenin is known as an important stemness regulatory pathway and several enriched signatures in mRRM2^+^-like subgroup referred to its potential activation. In this case, β-catenin was also examined. Consistently, both β-catenin protein itself and a group of β-catenin target genes presented higher expression level in the sorted RRM2^+^ iCCA cells than in RRM2^−^ iCCA cells (Fig. 7E-F). Comparably, the increased β-catenin level in RRM2^+^ cells mainly occurred in the cell nuclear fraction (Fig. 7G). Moreover, silencing β-catenin significantly reduced the cell malignancy features including colony formation, cell migration and spheroid formation in both iCCA cell lines (Fig S8A-D). When a stable form of β-catenin (with mutations of Ser33 and Ser37 to Ala) were overexpressed, the above cell malignancy features were significantly increased (Fig S9A-D). These data consistently demonstrated that the membrane RRM2-positive iCCA cells possessed malignant and stemness molecular features, and that Wnt/β-catenin signaling was activated in RRM2^+^ iCCA cells and contributed to their malignancy features. However, the activated Wnt/β-catenin signaling did not seem to further increase the RRM2^+^ iCCA cell populations (Fig S10).

RRM2 was identified as a key candidate related to iCCA cell malignancy and stemness based on its total mRNA level from transcriptome data and its total protein level from proteomics data (Fig. 1). Thus, the association of membrane RRM2 protein level and cytoplasmic RRM2 level was analyzed in iCCA cohort 4 (Fig S11). Overall, they were significantly positively correlated. Moreover, in membrane-RRM2 high iCCA tumors, the cytoplasmic RRM2 was also highly abundant, whereas in cytoplasm-RRM2 high iCCA tumors, the level of membrane RRM2 was scattered. This demonstrated the different distribution patterns between membrane RRM2 and cytoplasmic RRM2. Consistently, we classified iCCA patients into the RRM2^high^ group and the RRM2^low^ group based on the median cut-off of RRM2 mRNA level in iCCA cohorts 1–3 (Fig S12A). The differentially expressed genes between the two groups were used to perform GSEA analysis (Fig S12B). Among the top 20 identified signatures from GSEA analysis, several tumor malignancy and cell cycle related gene sets were significantly enriched in the RRM2^high^ group in cohort 1–3 (4–6 signatures for each category in each cohort) (Fig S12C). However, only 1–2 progenitor cell-related gene sets were enriched in the three cohorts. Comparable data were obtained when the tertile cut-off of RRM2 was performed and there were less progenitor cell-related signatures enriched in the RRM2^high^ group (Fig S13). Thus, compared to total RRM2 high iCCAs, membrane RRM2-positive iCCAs had much more malignant and stemness molecular features.

## Discussion

iCCA is a biliary tree-origin epithelial malignancy in liver that has very poor clinical outcomes. Here in this study, we have identified membrane RRM2-positive iCCAs representing a subpopulation of iCCA cells with severe malignant and stemness features. RRM2 is known as a small subunit in ribonucleotide reductases which participate in nucleotide metabolism and catalyze the conversion of nucleotides to deoxynucleotides, maintaining the dNTP pools for DNA biosynthesis and repair. Meanwhile, it is also considered to function in tumor progression as a regulator of some oncogenic processes. For these functions, RRM2 was thought to locate in the cytoplasm. Thus, our studies have discovered a new localization of RRM2 in cell membrane and a new role of RRM2 as a cell surface marker to enrich malignant iCCAs with CSC features.

Firstly, in iCCA, we revealed that RRM2 was highly expressed in iCCA tumors with the highly activated MS signature, and in the sorted iCCA cells positive for a stemness reporter. Silencing RRM2 in iCCA cells significantly reduced several tumor malignancy features in vivo and in vitro. These were consistent with the reported role of RRM2 in tumor progression in other cancers such as lung cancer, glioblastoma and retinoblastoma [20–23].

Secondly, RRM2 presented on the cell membrane of iCCA tumors and nearly 45.2% of iCCA patients had strong membrane RRM2 IHC staining (IHC score ≥ 6, iCCA cohort 4). Via a series of assays including immunofluorescence, cell fractionation, cell surface biotinylation/IP, the cell membrane localization of RRM2 was validated. Moreover, via protein sequence analysis and a group of molecular chemistry assays, we have, for the first time, revealed that RRM2 protein contained an unclassical long signal peptide (1-44aa) and a potential transmembrane domain (223-246aa). Both regions were essential for RRM2 trafficking to the cell membrane via the ER-Golgi pathway.

In addition, although the deletion of RRM2’s signal peptide (1-44aa) could largely reduce the cell membrane localization of RRM2, the mutation of VLA (42-44aa, the signal peptidase recognition site) did not. Thus, this long SP was critical for RRM2 membrane trafficking, but the SP cleavage of RRM2 was not necessary for this processing. This result is similar to several known ER processed proteins such as ApoM, CD18 and iron transporter protein Mx IRT1 [48–50]. These membrane proteins also retained their N-terminal SP for trafficking and membrane localization. Taken together, it appeared that there were three different conditions in terms of RRM2 localization. 1) RRM2’s unclassical SP was not recognized by signal recognition particle (SRP) complex, leading to the cytoplasm localization of RRM2. 2) RRM2 SP was recognized by SRP complex, which then directed RRM2 to the ER. In the ER, RRM2 was processed by signal peptidase and ended at the cell membrane as a short RRM2 form without SP. 3) RRM2 SP was recognized by SRP complex, whereas in the ER the SP of RRM2 was not processed by signal peptidase and resulted in the localization at the cell membrane as a whole length RRM2 form. Interestingly, the N-terminal sequence of RRM2 differs in various species. Although homology of 69-389aa in human RRM2 and mouse RRM2 is 96.3%, the homology of 1-68aa is only 69.2% [51]. Thus, it is interesting to further investigate whether RRM2’s cell membrane localization was unique to humans. It is also important to further delineate whether membrane RRM2 could also regulate dNTP levels, cell mitosis and cell cycle.

More significantly, we successfully sorted RRM2^+^ iCCA cells. The sorted membrane RRM2^+^ cells exhibited phenotypic features and molecular features of cancer malignancy and stemness. Either iCCA patients with strong membrane RRM2 staining, or mRRM2^+^-like iCCA patients based on the membrane RRM2 signature, had significantly worse prognosis. RRM2^+^ iCCA cells had higher level of stemness-related key factors such as OCT4, SOX2, Nanog and β-catenin. Nevertheless, the expression of these factors was not changed after RRM2 silencing (Data not shown). The relationship of RRM2 and Wnt/β-catenin has been reported before. RRM2 silencing inactivated β-catenin signaling by enhancing phosphorylation of glucose synthase kinase 3β, while RRM2 overexpression triggered Wnt/β-catenin pathway activation [24, 52]. However, it was also reported that RRM2 may act as an inhibitor of β-catenin downstream and that Wnt can relieve the RRM2-induced inhibitory effect on β-catenin-LEF/T cell factor (TCF) transcriptional activity by stimulating the phosphorylation at Ser20 of RRM2 [53]. Here in our data, β-catenin signaling was activated in membrane RRM2^+^ cells and contributed to the malignant features of membrane RRM2^+^ iCCA cells. In addition, the activated Wnt/β-catenin signaling did not seem to enhance the membrane RRM2-positve cell population. Thus, it would be worth to explore thoroughly the regulatory relationship of β-catenin signaling and RRM2 in the future.

Currently, RRM2 targeting strategies are mainly focused on inhibiting its enzyme activity or reducing gene expression levels. However, due to the important role of RRM2 in maintaining dNTP content, promoting DNA synthesis and regulating the cell cycle, targeting the total RRM2 pool may have severe side effects. We found that membrane localization of RRM2 was only present in iCCA tumor cells but not in normal bile duct cells, hepatocytes or other cells of the tumor microenvironment. Therefore, it is critical and necessary to continuously investigate whether membrane RRM2 acted primarily as biomarker for a group of most malignant iCCA cells with CSC features, or as an important functional regulator. If it is a biomarker, efforts are needed to decode the main molecular regulatory pathway in this population and seek the ideal molecular targets. If membrane RRM2 also functioned in promoting iCCA malignancy features, methods of targeting the membrane RRM2 specifically may hold the hope of blocking iCCA progression.

## Conclusion

Intrahepatic cholangiocarcinoma is a malignant tumor type with obvious heterogeneity features. In this study, RRM2 was identified as an important iCCA malignancy related factor due to its significant high level in iCCA patients with activated malignancy and stemness signatures and in iCCA cells positive for stemness reporter. Our thorough investigation further revealed RRM2 not only as a novel membrane protein with an unclassical SP and a potential transmembrane domain, but also as a membrane biomarker to enrich cells with malignancy and stemness features in iCCA. These results improved our understanding on the role of RRM2 in iCCA, and also indicated the heterogeneity of iCCA from a new perspective and paved the way of developing new methods of targeting iCCAs.

## Supplementary Information

Supplementary Material 1.

## Data Availability

Data are available in a public, open access repository. The mRNA profiling data of HCC cohort 1 and iCCA cohort 1 were available at GEO datasets of NCBI (GSE76297, https://www.ncbi.nlm.nih.gov/geo/query/acc.cgi?acc=GSE76297). The mRNA sequencing data of HCC cohort 2 and iCCA cohort 2 were available at the Cancer Genome Atlas (TCGA) portal (https://portal.gdc.cancer.gov). The mRNA transcriptome data and tumor proteome data of iCCA cohort 3 were obtained from Dr. Jia Fan (https://www.sciencedirect.com/science/article/pii/S1535610821006590?via%3Dihub). All data relevant to the study are included in the article or uploaded as online supplemental information.

## Abbreviations

ER: Endoplasmic reticulum
FFPE: Formalin-fixed paraffin-embedded
GSEA: Gene Set Enrichment Analysis
HCC: Hepatocellular carcinoma
IF: Immunofluorescence
IHC: Immunohistochemistry
IP: Immunoprecipitation
iCCA: Intrahepatic cholangiocarcinoma
MS signature: Malignancy & stemness signature
Mass Spec: Mass spectrometry
qRT-PCR: Quantitative reverse transcription polymerase chain reaction
RRM2: Ribonucleotide reductase subunit M2
RNR: Ribonucleotide reductase
SP: Signal peptide
TM: Transmembrane domain
TCGA: The Cancer Genome Atlas
