# Supplementary material for: Membrane RRM2-positive cells represent a malignant population with cancer stem cell features in intrahepatic cholangiocarcinoma
Source: J Exp Clin Cancer Res. 2024 Sep 6;43:255. doi: 10.1186/s13046-024-03174-w (PMC11378576; doi:10.1186/s13046-024-03174-w)
Supplement: Supplementary file 1 — Supplementary Material 1. [file 13046_2024_3174_MOESM1_ESM.pdf]

Supplementary Materials

Membrane RRM2-Positive Cells Represent a Malignant Population with Cancer Stem Cell Features in Intrahepatic Cholangiocarcinoma

Yongzhi Zhao[1,#], Shuting Xue[1,#], Danduo Wei[1], Jianjuan Zhang[1], Nachuan Zhang[1], Liping Mao[1], Niya Liu[1], Lei Zhao[2], Jianing Yan[1], Yifan Wang[1], Xiujun Cai[1], Saiyong Zhu[1], Stephanie Roessler[3], Junfang Ji[1,*.]

*Corresponding author. Email: junfangji@zju.edu.cn

This file includes:

- Figure legends……………………………………………Page 1 to 3
- Supplementary tables (n=5) …………………………Page 4 to 10
- Supplementary figures (n=13) ………………………Page 11 to 23

Figure Legends:

Fig S1. The effectiveness of MS signature and OS4-GFP reporter in HCC and the implication of OS4-GFP reporter system in HUCCT1 cells. (A)The information of HCC cohorts 1-2 and the hierarchical clustering analysis based on the activation level of MS signature. HCC patients were divided into MS^{High}， MS^{Mid} and MS^{Low} groups. Hazard ratio of overall survival of MS^{High} group and MS^{Low} group were shown. Log-rank test was performed. (B) Relative expression level of EpCAM and CD24 in MS^{High} group and MS^{Low} group in HCC cohorts 1-2. (C) GFP fluorescence analysis of OS4-GFP^+ Huh7 and OS4-GFP^- Huh7 cells by flow cytometry. Percentage of Huh7 GFP^+ cells at day 0 or day 3 after cell sorting was quantified. (D) Expression levels of HCC malignancy and stemness-related genes were determined by RT-qPCR (mean ± SD) in OS4-GFP^+ and OS4-GFP^- Huh7 cells at day 0 after cell sorting. (E) The GFP fluorescence level of GFP^+ HUCCT1 and GFP^- HUCCT1 cells was analyzed by flow cytometry. Percentage of GFP^+ cells at day 0 and at the day 5 after cell sorting was quantified. (F) Spheroid formation was performed and compared between OS4-GFP^+ HUCCT1 cells and OS4-GFP^- HUCCT1 cells. Spheroids with diameter ≥ 50μm were counted. (G) OCT4, NANOG, SOX2, and RRM2 levels in OS4-GFP^+ HUCCT1 cells and OS4-GFP^- HUCCT1 cells. Colony formation and cell

migration were performed and compared between OS4-GFP^{+} HUCCT1 cells and OS4-GFP^{-} HUCCT1 cells. (B, C, E, F, G) The Student's *t*-test was used.

Fig S2. Hierarchical clustering analysis of MS^{High} & MS^{Low} patients and OS4-GFP^{+} & OS4-GFP^{-} cells. (A-C) Hierarchical clustering analysis of MS^{High} and MS^{Low} patients using genes with significant two-fold higher expression in MS^{High} group vs. MS^{Low} group in each iCCA cohort (146 genes in iCCA cohort 1, 503 genes in iCCA cohort 2, 252 genes in iCCA cohort 3). (D) Heatmap of 301 proteins with significant two-fold higher expression in OS4-GFP^{+} vs. OS4-GFP^{-} RBE cells.

Fig S3. IHC staining scores of RRM2 in iCCA tumors, normal bile duct and hepatocytes. The Student's *t*-test was performed.

Fig S4. The potential transmembrane domain of RRM2 was essential for RRM2 cell membrane localization. (A) The expression of RRM2-Flag, β-catenin and EGFR in RBE and HUCCT1 cells with MG132 and BafA1 treatment. (B) The expression of RRM2 with 36-38aa deletion (ALS del) or 42-44 aa deletion (VLA del) in RBE and HUCCT1 cells. (C) The expression of RRM2 with 223-246aa truncation or mutations of hydrophobic amino acids in 223-246aa region was detected by anti-Flag in HUCCT1 cells. Confocal microscopy images of exogenous RRM2 detected by anti-Flag in HUCCT1 cells. Yellow arrows indicate the cell membrane localization of RRM2. Percentage of RRM2 cell membrane localization was measured (mean ± SD). The Student's *t*-test was used.

Fig S5. RRM2 membrane location by flow cytometry and immunofluorescence. (A) Membrane RRM2 staining in RBE and HUCCT1 cells was determined by flow cytometry using the antibody which recognized 1-111aa of RRM2, with or without GCA treatment. Grey line, no staining; red line, DSMO treatment; blue line, GCA treatment. (B) Confocal microscopy images of endogenous RRM2 and Glogi maker protein GOLPH2, and their co-localization in RBE and HUCCT1 cells.

Fig S6. Cell proliferation and DNA replication in RRM2^{+} iCCA cells and RRM2^{-} iCCA cells. (A) Cell viability was performed and compared in sorted RRM2^{+} and RRM2^{-} cells from both RBE and HUCCT1 iCCA cell lines. Two-way ANOVA analysis was used. (B) Immunofluorescence for EdU labeling in sorted RRM2^{+} and RRM2^{-} cells from both RBE and HUCCT1 iCCA cell lines.. Percentage of EdU-positive cells was measured (mean ± SD). The Student's *t*-test was used.

Fig S7. The top 20 enriched signatures in mRRM2^{+} -like patients in iCCA cohorts 1-3. Red bars represent malignancy-related signatures, orange bars represent progenitor cell-related signatures, dark grey bars represent cell cycle/cell division-related signatures and light grey

bars represent other signatures.

Fig S8. β-catenin silencing suppressed iCCA malignancy features. (A) Silencing efficiency of β-catenin (gene name: CTNNB1) siRNAs in RBE and HUCCT1 cells was evaluated by RT-qPCR and western blot. (B-D) colony formation(B), cell migration(C) and spheroid formation(D) were performed in iCCA cells transfected with siCtrl or siCTNNB1. Spheroids with diameter ≥ 50μm were counted. (B, C, D) The Student's *t*-test was used.

Fig S9. Overexpression of β-catenin promoted iCCA malignancy features. (A) Overexpression of mutant β-catenin in RBE and HUCCT1 cells was evaluated by western blot. (B-D) colony formation(B), cell migration(C) and spheroid formation(D) were performed in iCCA cells transfected with Ctrl-HA or β-catenin^{mut}-HA. Spheroids with diameter ≥ 50μm were counted. (B, C, D) The Student's *t*-test was used.

Fig S10. Membrane RRM2-positive populations after altering Wnt/β-catenin activation. (A) RBE and HUCCT1 cells were transfected with siCTNNB1 and membrane RRM2 staining was determined by flow cytometry. (B) RBE and HUCCT1 cells were transfected with β-catenin^{mut} and membrane RRM2 staining was determined by flow cytometry. (C) RBE and HUCCT1 cells were treated with LiCl. The protein expression β-catenin was examined with Western blot and membrane RRM2 staining was determined by flow cytometry.

Fig S11. The association of RRM2 cell membrane localization and cytoplasm localization. The spearmen correlation of RRM2 cell membrane staining scores and RRM2 cytoplasm staining scores in iCCA cohort 4.

Fig S12. The molecular signatures enriched in RRM2^{high} iCCA group based on the median cut-off of RRM2. (A) iCCAs patients were divided into two subgroups based on the median cut-off of RRM2 (RRM2^{high}, RRM2^{low}). (B) Genes with differential expression between RRM2^{high} and RRM2^{low} subgroups. Genes with significant altered expression were used to GSEA analysis. (C) The top 20 enriched signatures in RRM2^{high} group of iCCA cohorts 1-3. (D) Kaplan-Meier analysis of overall survival for RRM2^{High} subgroup and RRM2^{Low} subgroup in iCCA cohorts 1-3.

Fig S13. The molecular signatures enriched in RRM2^{high} iCCA group based on the tertile cut-off of RRM2. (A) The expression of RRM2 in different cohorts. iCCAs patients were divided into three subgroups based on the level of RRM2 (RRM2^{high}, RRM2^{mid}, RRM2^{low}). (B) Genes with differential expression between RRM2^{high} and RRM2^{low} subgroups. Genes with significant altered expression were used to GSEA analysis. (C) The top 20 enriched signatures in RRM2^{high} group of iCCA cohorts 1-3. (D) Kaplan-Meier analysis of overall survival for RRM2^{High} subgroup, RRM2^{Mid} subgroup and RRM2^{Low} subgroup in iCCA cohorts 1-3.

Table S1. Primers and Oligos used in this study

Primers	Sequence
	**Primers for plasmids construction**
Flag-RRM2	F: AAGGAAAAAAGCGGCCGCTCTCTCCCTCCGTGTCCCG<br>R: TGCTCTAGATTAGAAGTCAGCATCCAAGGTA
RRM2-Flag	F: AAGGAAAAAAGCGGCCGCATGCTCTCCCTCCGTGTC<br>R: TGCTCTAGAGAAGTCAGCATCCAAGGTAAAAG
RRM2 Δ1-38	F: AAGGAAAAAAGCGGCCGCATGGGGACCCGCGTCCTGGC<br>R: TGCTCTAGAGAAGTCAGCATCCAAGGTAAAAG
RRM2 Δ1-44	F: AAGGAAAAAAGCGGCCGCATGAGCAAGACCGCGAGGAGGATC<br>R: TGCTCTAGAGAAGTCAGCATCCAAGGTAAAAG
RRM2 ALS/DDD	F1: AAGGAAAAAAGCGGCCGCATGCTCTCCCTCCGTGTC<br>R1: TGCTCTAGAGAAGTCAGCATCCAAGGTAAAAG<br>F2: CAAGGAGAACACGCCGCCGGACGACGACGGGACCCGCGTCCTGG<br>R2: CCAGGACGCGGGTCCCGTCGTCGTCCGGCGGCGTGTTCTCCTTG
RRM2 VLA/DDD	F1: AAGGAAAAAAGCGGCCGCATGCTCTCCCTCCGTGTC<br>R1: TGCTCTAGAGAAGTCAGCATCCAAGGTAAAAG<br>F2: GCCCTGAGCGGGACCCGCGACGACGACAGCAAGACCGCGAGGAGG<br>R2: CCTCCTCGCGGTCTTGCTGTCGTCGTCGCGGGTCCCGCTCAGGGC
RRM2 double DDD	F1: AAGGAAAAAAGCGGCCGCATGCTCTCCCTCCGTGTC<br>R1: TGCTCTAGAGAAGTCAGCATCCAAGGTAAAAG<br>F2: GACGACGACGGGACCCGCGACGACGACAGCAAGACCGCGAGGAGG<br>R2: CCTCCTCGCGGTCTTGCTGTCGTCGTCGCGGGTCCCGTCGTCGTC
RRM2 Δ223-246	F1: AAGGAAAAAAGCGGCCGCATGCTCTCCCTCCGTGTC<br>R1: TGCTCTAGAGAAGTCAGCATCCAAGGTAAAAG<br>F2: GAGGCTACCTATGGTAAGAAACGAGGACTG<br>R2: CAGTCCTCGTTTCTTACCATAGGTAGCCTC
RRM2 12G	F1: AAGGAAAAAAGCGGCCGCATGCTCTCCCTCCGTGTC<br>R1: TGCTCTAGAGAAGTCAGCATCCAAGGTAAAAG<br>F2: CGGCACCACCACGTTCACCATAGGTAGCCTC<br>R2: TCGGGTGGTGGTGGTAAGAAACGAGGACTGAT<br>F3: GAGGCTACCTATGGTGAACGTGGTGGTGCCGGTGCTGCAGGGGAAGG CGGTGGTGGTTCCGGTTCTGGTGCGTCGGGTGGTGGTGGT<br>R3: ACCACCACCACCCGACGCACCAGAACCGGAACCACCACCGCCTTCCC CTGCAGCACCGGCACCACCACGTTCACCATAGGTAGCCTC
4×OCT4/SOX2 binding elements	F: TAATTTTGCATTACAATGTTTTGCATTACAATGTTTTGCATTACAATGTTTT GCATTACAATG

	R: CCGGCATTGTAATGCAAAACATTGTAATGCAAAACATTGTAATGCAAAACA TTGTAATGCAAAAT
CTNNB1	F: GTACAAAAAAGCAGGCAGCTAGCATGTATCCATATGATGTTCCAGATTATG CTACCGG<br>R: AACTAGAATGCAGCGGCCGCTTACAGGTCAGTATCAAACCAGGCCAGC

Primers for Real-time PCR

RRM2	F: CACGGAGCCGAAAACTAAAGC<br>R: TCTGCCTTCTTATACATCTGCCA
OCT4	F: CTTGAATCCCGAATGGAAAGGG<br>R: GTGTATATCCCAGGGTGATCCTC
SOX2	F: TGGACAGTTACGCGCACAT<br>R: CGAGTAGGACATGCTGTAGGT
NANOG	F: CCCCAGCCTTTACTCTTCCTA<br>R: CCAGGTTGAATTGTTCCAGGTC
MYC	F: GTCAAGAGGCGAACACACAAC<br>R: TTGGACGGACAGGATGTATGC
CCND1	F: CCTCTGTGCCACAGATG<br>R: GGGTCACACTTGATCACTC
AXIN2	F: TATCCAGTGATGCGCTGACG<br>R: TTACTGCCCACACGATAAGG
DKK1	F: TCCCCTGTGATTGCAGTAAA<br>R: TCCAAGAGATCCTTGCGTTC
LGR5	F: TCAGTCAGCTGCTCCCGAAT<br>R: CGTTTCCCGCAAGACGTAAC
CLND1	F: CCTCCTGGGAGTGATAGCAAT<br>R: GGCAACTAAAATAGCCAGACCT
MMP14	F: CGAGGTGCCCTATGCCTAC<br>R: CTCGGCAGAGTCAAAGTGG
PPARD	F: CAGGGCTGACTGCAAACGA<br>R: CTGCCACAATGTCTCGATGTC
PLAU	F: CTGTCACCTACGTGTGTGGAG<br>R: TGAGCGACCCAGGTAGACG
CDKN2A	F: ATGGAGCCTTCGGCTGACT<br>R: GTAACTATTCGGTGCGTTGGG
LAMC2	F: GACAAACTGGTAATGGATTCCGC<br>R: TTCTCTGTGCCGGTAAAAGCC
BRIC5	F: TCAAGGACCACCGCATCTCT

	R: ATGTTCCTCTATGGGGTCGTC
ENC1	F: GCCAGCCATCTATCTCATGGA<br>R: GGTTACCACACCGTCATTCTG
ID2	F: GCTATACAACATGAACGACTGCT<br>R: AATAGTGGGATGCGAGTCCAG
CD24	F: CTCCTACCCACGCAGATTTATTC<br>R: AGAGTGAGACCACGAAGAGAC
CD44	F: GCAAACACAACCTCTGGTCC<br>R: CCCACACCTTCTTCGACTG
EpCAM	F: GCTCTGAGCGAGTGAGAACC<br>R: ACGCGTTGTGATCTCCTTCT
KLF4	F: GGGAGAAGACACTGCGTCAA<br>R: GGAAGTCGCTTCATGTGGGA
DCLK1	F: TGAACAAGAAGACGGCTCACTCC<br>R: GCTGGTGGGTGATGGACTTGG
OV6	F: GGGCAATGGATTGGTCATCCT<br>R: TGCAGCCTGTACTTGTCCG
CK19	F: AACGGCGAGCTAGAGGTGA<br>R: GGATGGTCGTGTAGTAGTGGC
CD90	F: ATCGCTCTCCTGCTAACAGTC<br>R: CTCGTACTGGATGGGTGAACT
UGT2B7	F: GATCCCAACAACTCATCCGCT<br>R: CAGCAGCTCACTACAGGGAA7
CTNNB1	F: AAAGCGGCTGTTAGTCACTGG<br>R: CGAGTCATTGCATACTGTCCAT
U6	F: CTCGCTTCGGCAGCACA<br>R: AACGCTTCACGAATTTGCGT
GAPDH	F: ACCCACTCCTCCACCTTTGAC<br>R: TGTTGCTGTAGCCAAATTCGTT
18S	F: GACTCAACACGGGAAACCTC<br>R: AGCATGCCAGAGTCTCGTTC

SiRNA sequences

RRM2 siRNA#1	CCCATCGAGTACCATGATA
RRM2 siRNA#2	GGTGGAGCGATTTAGCCAA
CTNNB1 siRNA#1	ACGACTAGTTCAGTTGCTT
CTNNB1 siRNA#2	GTGCTATCTGTCTGCTCTA

Table S2. Clinical Characteristics of the MS^{high} and MS^{low} subgroups in iCCA Cohort 1.

Clinical Variable	MS^{high} n=35	MS^{low} n=22	P value
Gender			
Female	8	8	0.23[a]
Male	24	11	
Missing	3	3	
Age-year			
Median (range)	60 (39-76)	62 (40-79)	0.35[b]
ALT (U/L)			
Normal (≤55)	24	17	0.29[a]
Abnormal (>55)	8	2	
Missing value	3	3	
CA19-9 (U/mL)			
Normal (≤40)	14	9	1.00[a]
Abnormal (>40)	12	9	
Missing value	9	4	
Overall survival-month			
Median	10.2	15.6	0.09[c]
Range	0.2-29.6	0.9-29.9	

[a], Fisher's exact test; [b], Unpaired t-test; [c], Log-rank test

Table S3. Clinical Characteristics of the MS^{high} and MS^{low} subgroups in iCCA Cohort 2.

Clinical Variable	MS^{high} n=14	MS^{low} n=10	P value
Gender			
Female	7	5	1.00[a]
Male	7	5	
Age-year			
Median (range)	61 (31-82)	69.5 (29-77)	0.86[b]
TNM stage			
I-II	13	8	0.55[a]
III-IV	1	2	
Overall survival-month			
Median	24.0	28.0	**0.04**[c]
Range	3.3-65.9	1.7-53.8	

[a], Fisher's exact test; [b], Unpaired t-test; [c], Log-rank test

Table S4. Clinical Characteristics of the MS^{high} and MS^{low} subgroups in iCCA Cohort 3.

Clinical Variable	MS^{high} n=96	MS^{low} n=77	P value
Gender			
Female	40	57	0.53[a]
Male	56	71	
Age-year			
Median (range)	61.5 (30-82)	62 (27-86)	0.84[b]
ALT (U/L)			
Normal (≤55)	85	73	0.18[a]
Abnormal (>55)	11	5	
Albumin (g/L)			
Normal (≥35)	94	74	0.66[a]
Abnormal (<35)	2	3	
CA19-9 (U/mL)			
Normal (≤40)	42	40	0.29[a]
Abnormal (>40)	54	37	
CEA (ng/mL)			
Normal (≤5)	70	65	0.10[a]
Abnormal (>5)	26	12	
TNM stage			
I-II	59	57	0.10[a]
III-IV	37	20	
Tumor size (cm)			
≤3	10	14	0.18[a]
>3	86	63	
Intrahepatic metastasis			
Yes	41	18	**<0.01[a]**
No	55	59	
Distal metastasis			
Yes	7	1	0.08[a]
No	89	76	
Vascular invasion			
Yes	51	25	**<0.01[a]**
No	45	52	
Overall survival-month			
Median	18.7	25.7	**<0.001[c]**
Range	1.4-53.0	2.4-60.2	

[a], Fisher's exact test; [b], Unpaired t-test; [c], Log-rank test

Table S5. Clinical Characteristics of the RRM2 mem^{high}, mem^{low}, $cyto^{high}$ and $cyto^{low}$ subgroups in iCCA Cohort 4.

Clinical Variable	Membrane RRM2 staining score			Cytosol RRM2 staining score		
	mem^{high} Score ≥ 6 n=14	mem^{low} Score ≤ 5 n=17	p-value	$cyto^{high}$ Score ≥ 4 n=15	$cyto^{low}$ Score ≤ 3 n=16	P value
Gender						
Female	2	8	0.05[a]	4	6	0.45[a]
Male	12	7		11	8	
Missing value	0	2		0	2	
Age-year						
Median (range)	64 (45-72)	59 (43-77)	0.32[b]	64 (45-77)	59 (43-71)	0.10[b]
ALT (U/L)						
Normal (≤55)	12	10	0.39[a]	12	10	0.68[a]
Abnormal (>55)	2	5		3	4	
Missing value	0	2		0	2	
Albumin (g/L)						
Normal (≥35)	13	11	0.33[a]	13	11	0.65[a]
Abnormal (<35)	1	4		2	3	
Missing value	0	2		0	2	
AFP (ng/ml)						
Normal (≤20)	11	12	1.00[a]	13	10	0.39[a]
Abnormal (>20)	3	3		2	4	
Missing value	0	2		0	2	
CA19-9 (U/ml)						
Normal (≤40)	6	5	0.71[a]	6	5	1.00[a]
Abnormal (>40)	8	10		9	9	
Missing value	0	2		0	2	
CEA (ng/ml)						
Normal (≤5)	10	7	0.26[a]	9	8	1.00[a]
Abnormal (>5)	4	8		6	6	
Missing value	0	2		0	2	
TNM Stage						
I-II	3	7	0.24[a]	5	5	1.00[a]
III-IV	10	7		9	8	
Missing value	1	3		1	3	
Tumor size (cm)						
≤3	1	2	1.00[a]	2	1	0.59[a]
>3	9	11		9	11	
Missing value	4	4		4	4	
Overall survival-month						
Median	16.2	29.8	**0.04[c]**	29.7	18.4	0.82[c]
Range	1.8-36.9	7.1-36.9		3.5-36.9	1.8-36.9	

[a], Fisher's exact test; [b], Unpaired t-test; [c], Log-rank test

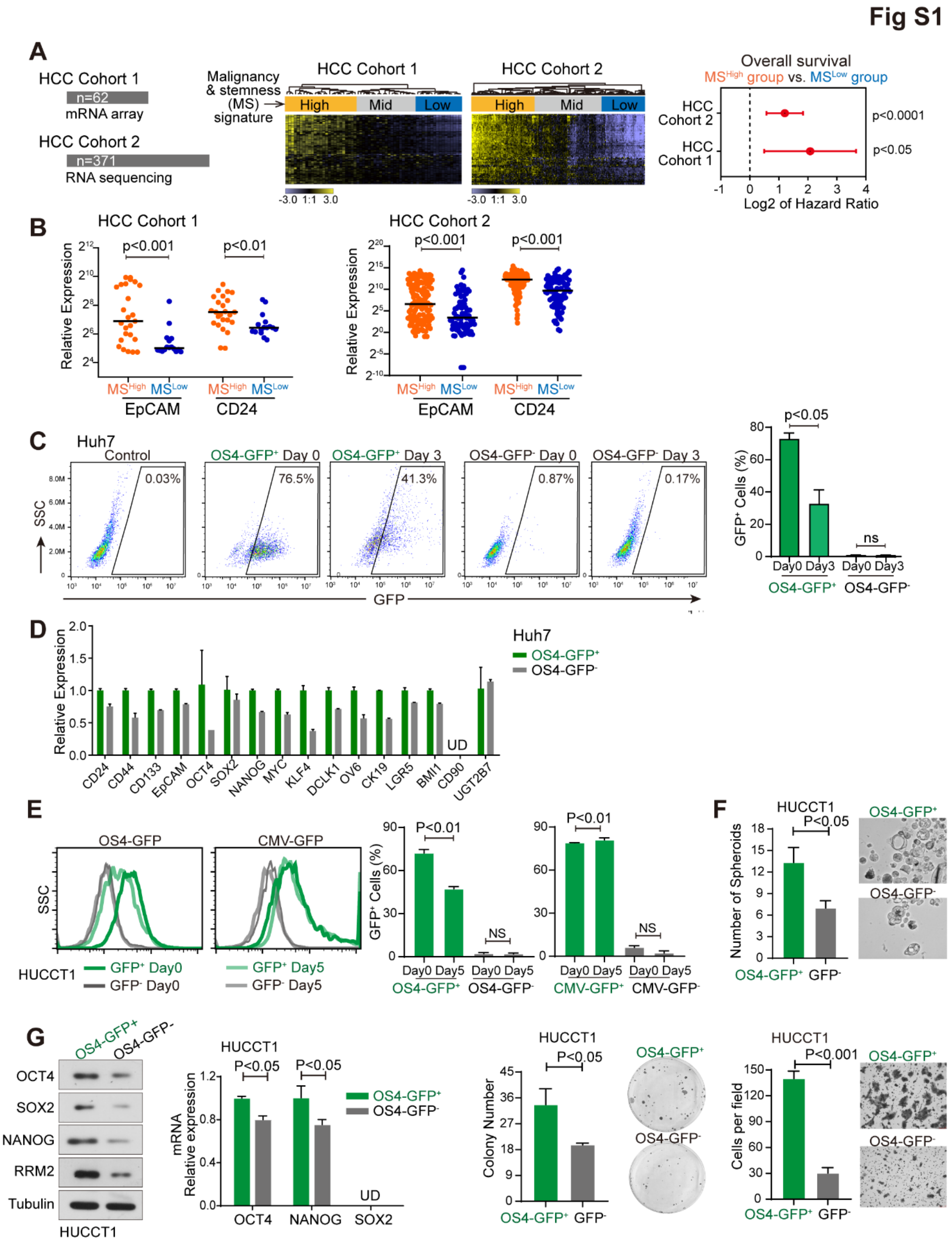

Fig S1. The effectiveness of MS signature and OS4-GFP reporter in HCC and the implication of OS4-GFP reporter system in HUCCT1 cells.

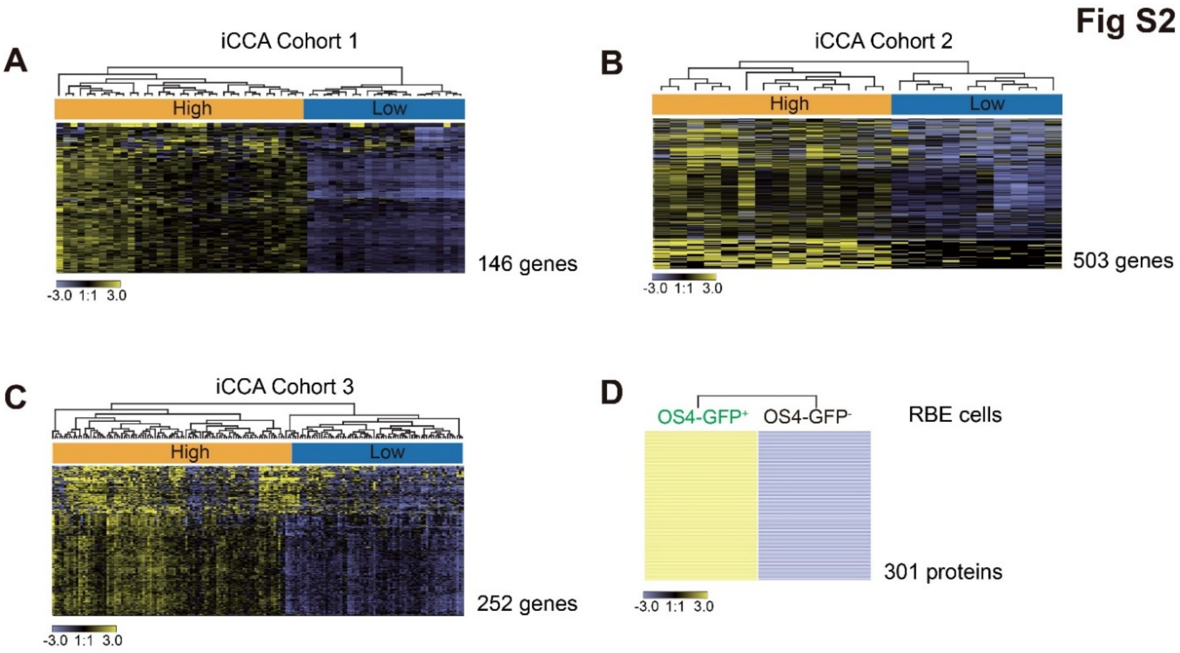

Fig S2. Hierarchical clustering analysis of MS^{High} & MS^{Low} patients and OS4-GFP^{+} & OS4-GFP^{-} cells.

Fig S3

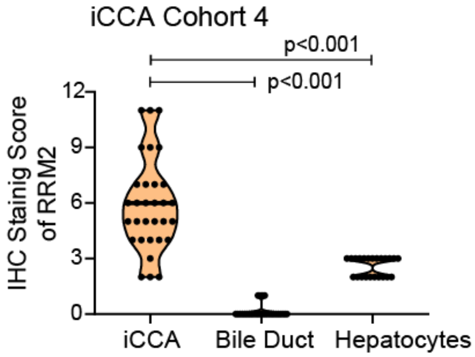

Fig S3. IHC staining scores of RRM2 in iCCA tumors, normal bile duct and hepatocytes.

Fig S4

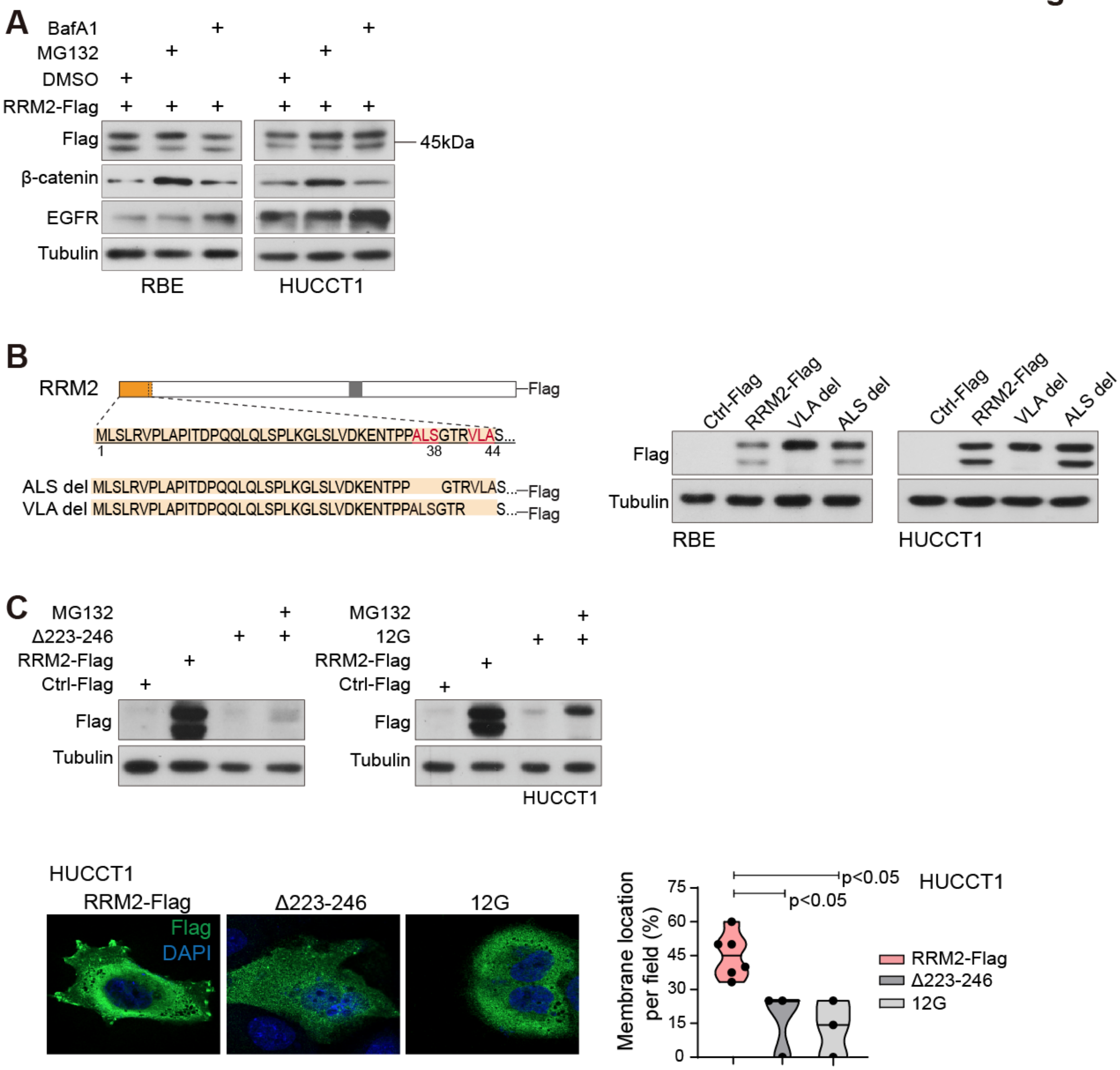

Fig S4. The potential transmembrane domain of RRM2 was essential for RRM2 cell membrane localization.

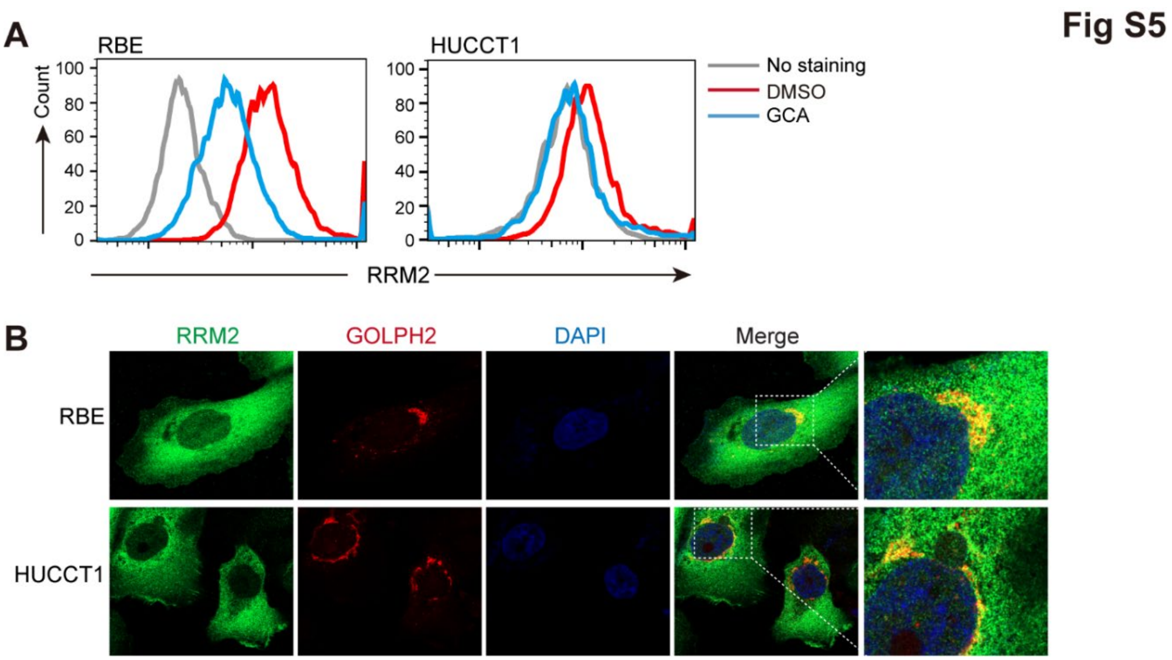

Fig S5. **RRM2 membrane location by flow cytometry and immunofluorescence.**

Fig S6

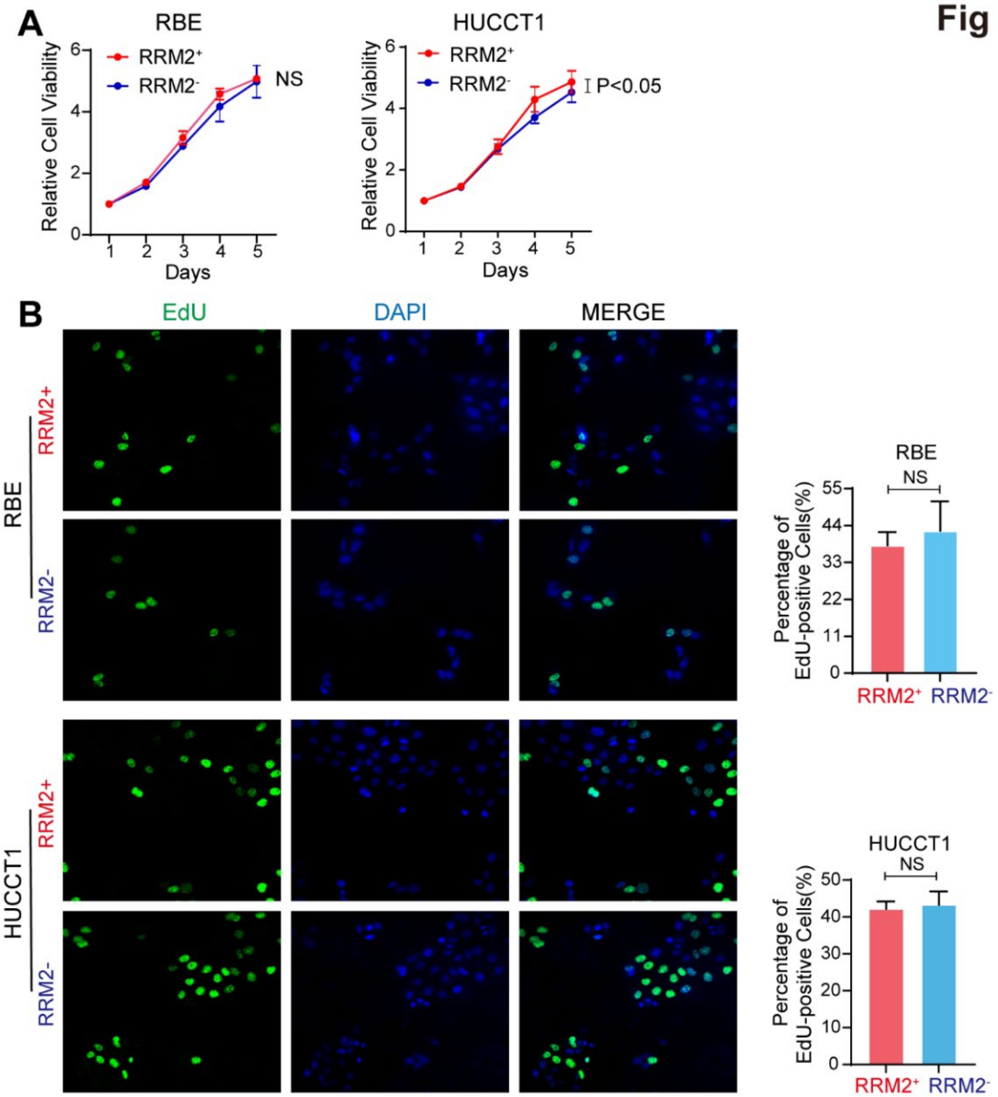

Fig S6. Cell proliferation and DNA replication in RRM2^{+} iCCA cells and RRM2^{-} iCCA cells.

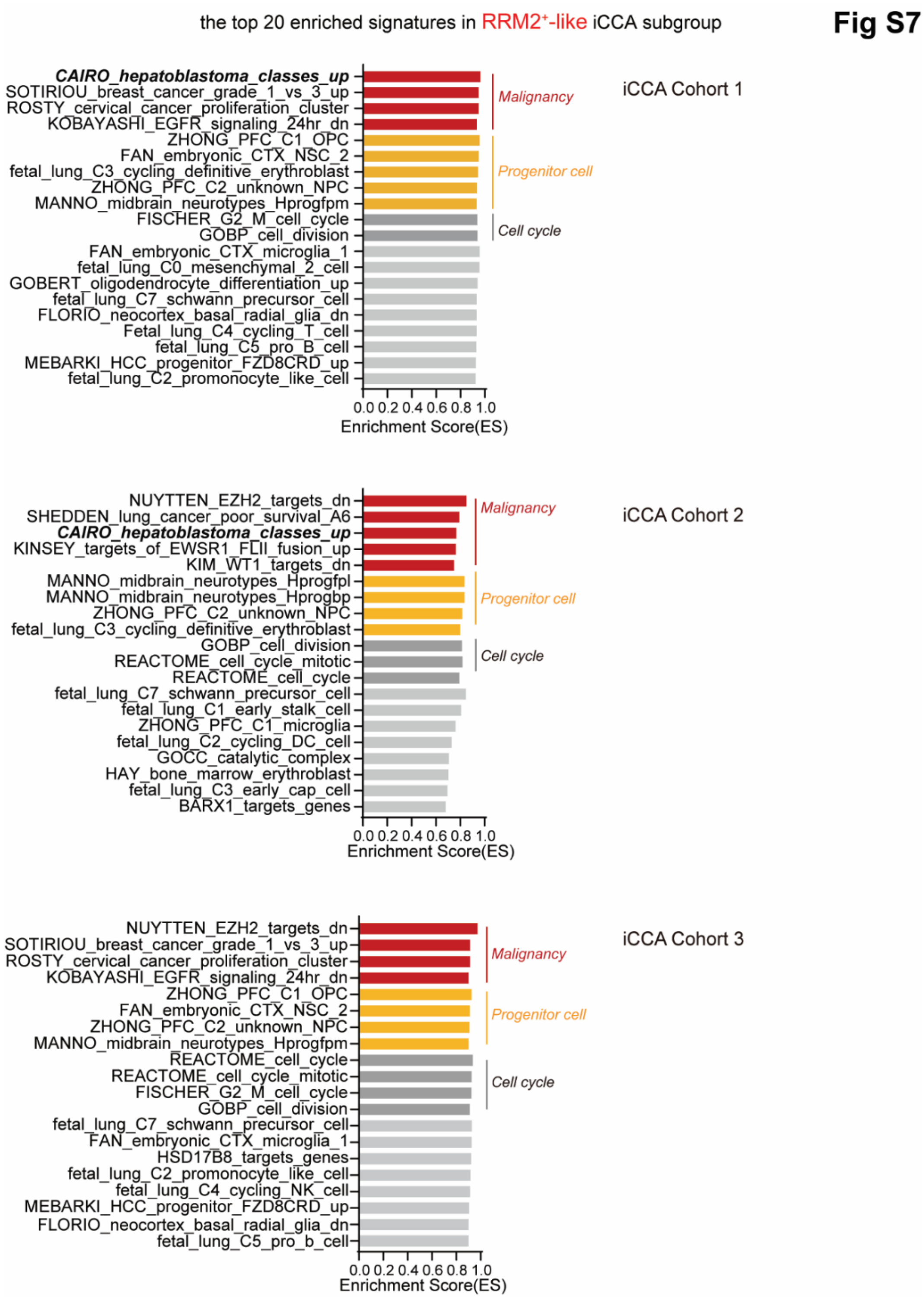

Fig S7. The top 20 enriched signatures in mRRM2⁺ -like patients in iCCA cohorts 1-3.

Fig S8

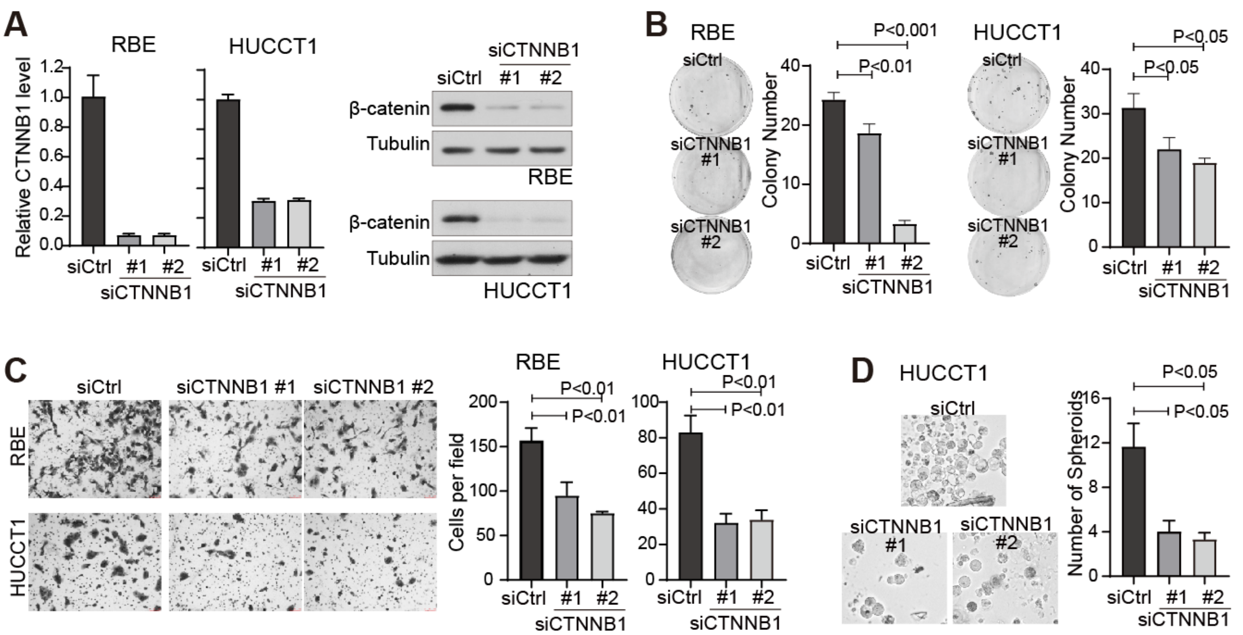

Fig S8. β-catenin silencing suppressed iCCA malignancy features.

Fig S9

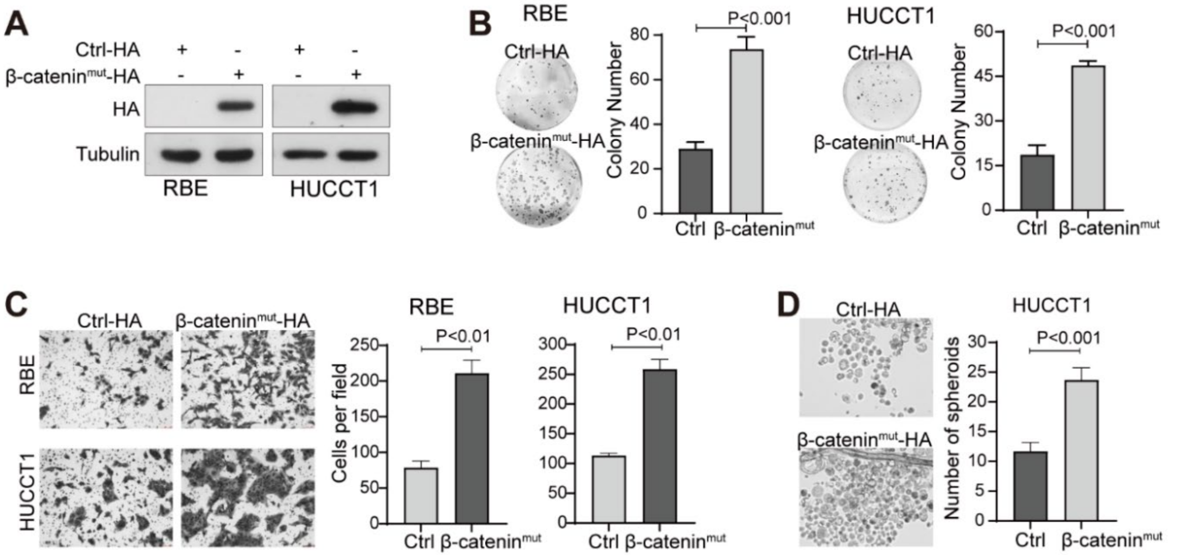

Fig S9. Overexpression of β-catenin promoted iCCA malignancy features.

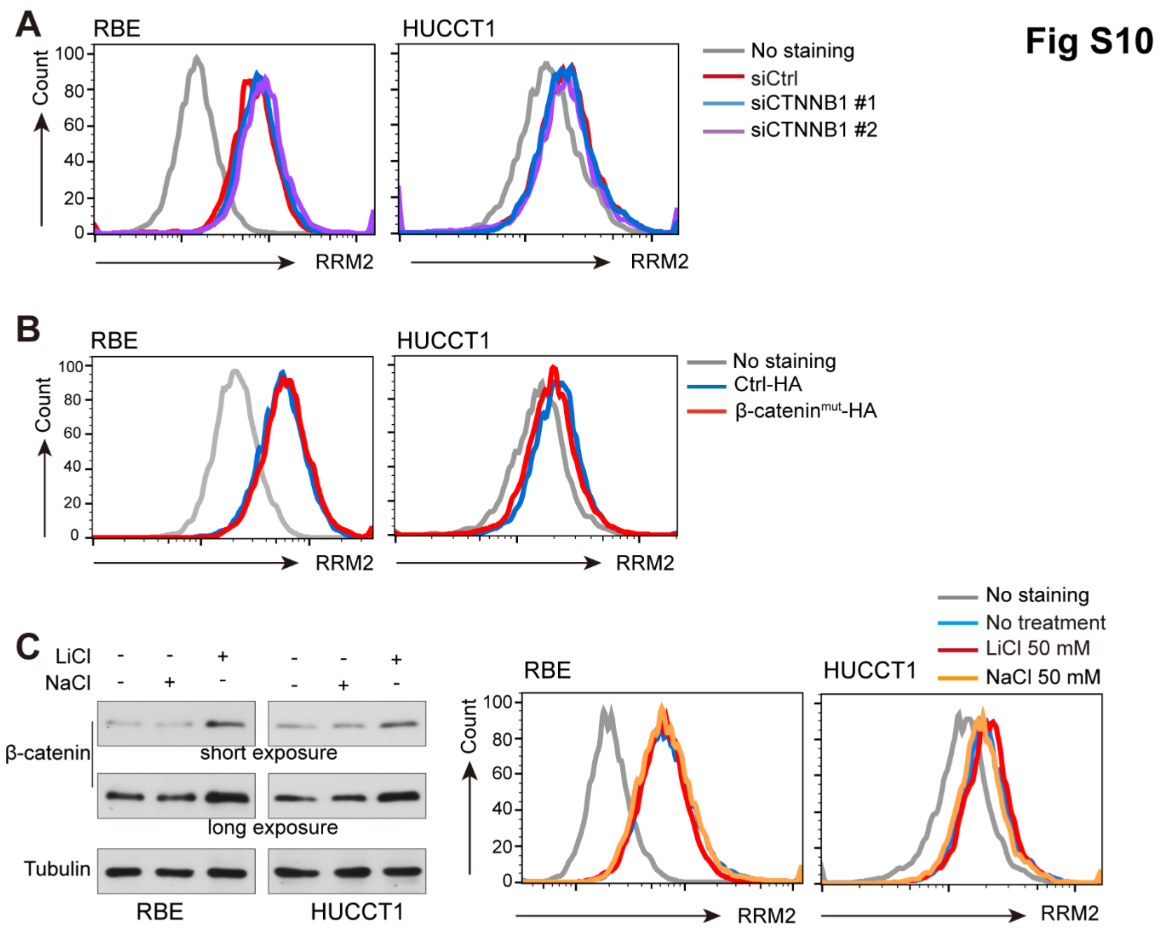

Fig S10. Membrane RRM2-positive populations after altering Wnt/β-catenin activation.

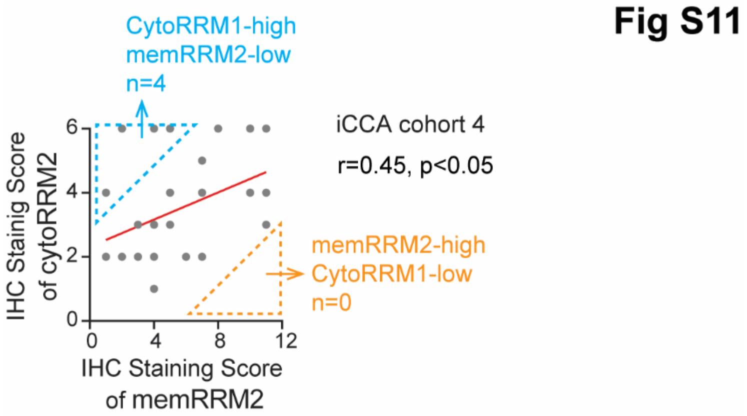

Fig S11. The association of RRM2 cell membrane localization and cytoplasm localization.

Fig S12

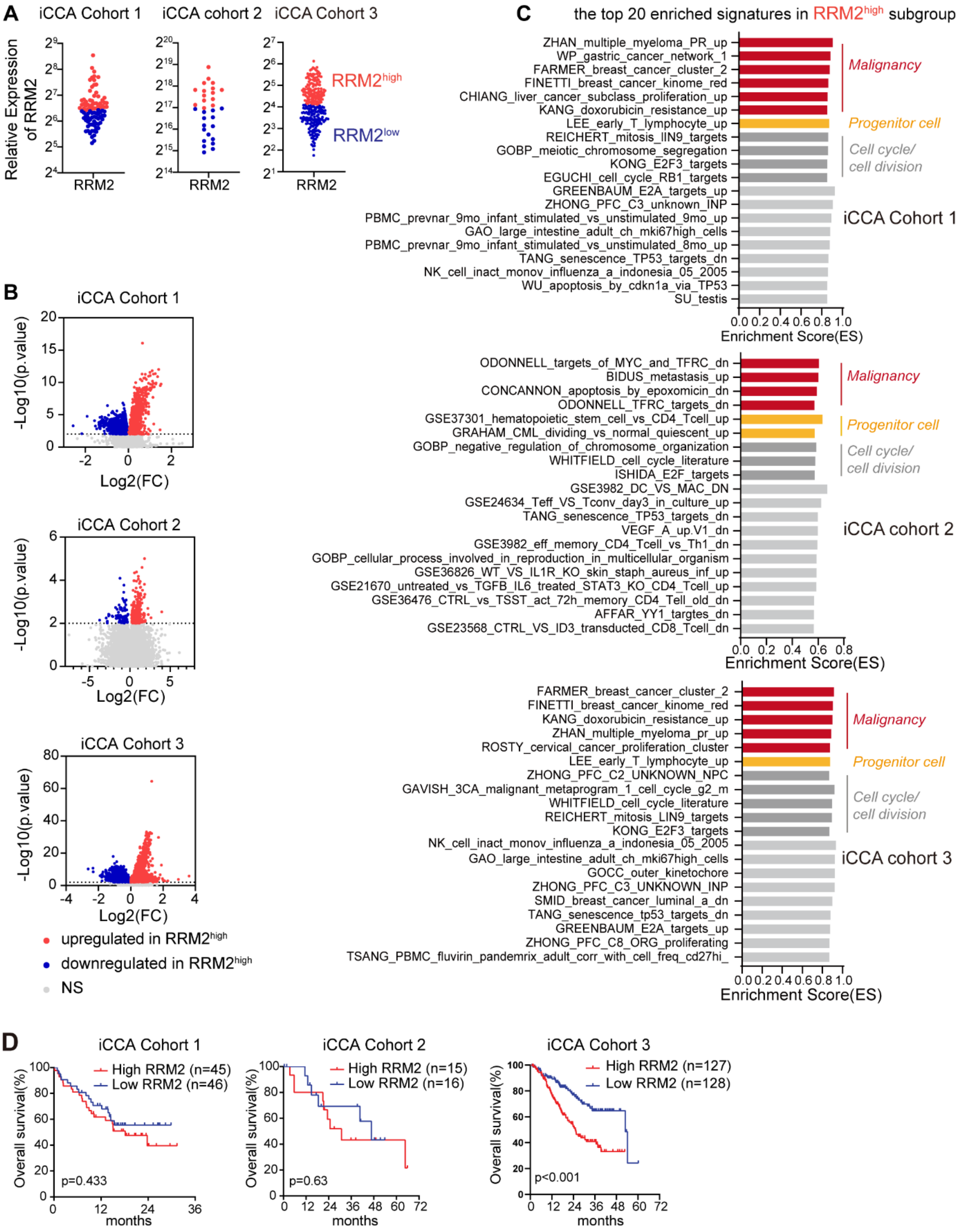

Fig S12. The molecular signatures enriched in RRM2^{high} iCCA group based on the median cut-off of RRM2.

Fig S13

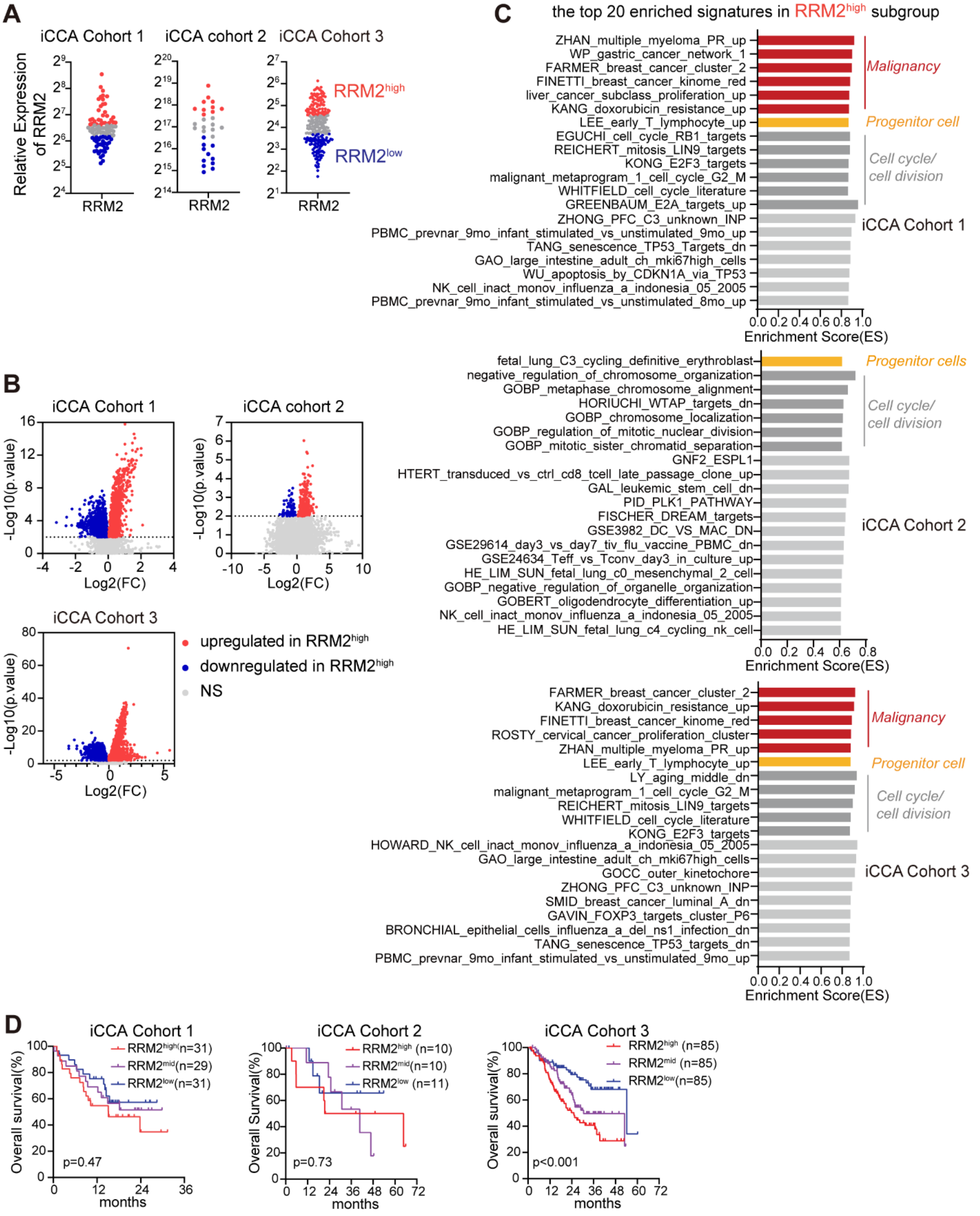

Fig S13. The molecular signatures enriched in RRM2^{high} iCCA group based on the tertile cut-off of RRM2.
